# Malformin A1–mediated cytotoxicity in ovarian cancer cells occurs through pyroptosis and autophagy

**DOI:** 10.1002/2211-5463.70296

**Published:** 2026-07-04

**Authors:** Nada Abdullah Hassan, Ikram A. Burney, Shika Hanif Malgundkar, Siva Ramamoorthy, Ressin Varghese, Mohamed A. Al‐Kindi, Hamad Al Riyami, Shadia Al Bahlani, Sergey Dobretsov, Syed Imran Hassan, Benjamin K. Tsang, Yahya Tamimi

**Affiliations:** ^1^ Biochemistry Department, College of Medicine and Health Sciences Sultan Qaboos University Muscat Sultanate of Oman; ^2^ Women Health Program, Sultan Qaboos Comprehensive Cancer Care and Research Center University Medical City Muscat Sultanate of Oman; ^3^ School of Bio Sciences and Technology Vellore Institute of Technology India; ^4^ Pathology Department, College of Medicine and Health Sciences Sultan Qaboos University Muscat Sultanate of Oman; ^5^ Genetics Department, College of Medicine and Health Sciences Sultan Qaboos University Muscat Sultanate of Oman; ^6^ Allied Health Sciences, College of Medicine and Health Sciences Sultan Qaboos University Muscat Sultanate of Oman; ^7^ Department of Marine Science & Fisheries, College of Agricultural & Marine Sciences Sultan Qaboos University Muscat Oman; ^8^ Centre of Excellence in Marine Biotechnology Sultan Qaboos University Muscat Oman; ^9^ Department of Chemistry, College of Science Sultan Qaboos University Muscat Oman; ^10^ Chronic Disease Program Ottawa Hospital Research Institute Canada

**Keywords:** autophagy, cytoskeleton, Malformin A1, ovarian cancer, pyroptosis

## Abstract

Cytoskeletal proteins play a crucial role in providing mechanical support and regulating key cellular processes such as cell proliferation, migration, and invasion. Cytoskeletal damage has been increasingly regarded as a contributing factor in impairing these cellular processes in cancer. Moreover, induction of cell death pathways has been linked to cytoskeletal destabilization. However, the effect of cytoskeletal disruption on cell death mechanisms in ovarian cancer (OC) remains elusive. Several natural compounds have been demonstrated to initiate cytoskeletal destabilization as a mechanism to promote cell death. We have previously shown that one such natural compound derived from marine sources, Malformin A1 (MA1), exhibits high toxicity toward both cisplatin‐sensitive (A2780S) and cisplatin‐resistant (A2780CP) OC cell lines. Thus, here we evaluate the impact of cytoskeletal destabilization by MA1 treatment on OC cell death by analyzing the expression levels of apoptosis, autophagy, and DNA damage‐related genes. Our findings show MA1 treatment significantly downregulated key cytoskeletal proteins while also decreasing the expression of pro‐apoptotic markers, suggesting alternative cell death mechanisms. Autophagy‐related analyses demonstrated enhanced LC3BI to LC3BII processing, indicating autophagy activation with elevated γ‐H2AX levels confirming substantial DNA damage in MA1‐treated cells. Notably, MA1 was able to induce pyroptotic cell death, as evidenced by increased caspase‐1 expression. Moreover, molecular docking analysis revealed that MA1 displayed the strongest binding affinity for vimentin, GAPDH, and β‐tubulin, providing mechanistic insights into its ability to disrupt cytoskeletal integrity and induce nonapoptotic cell death through multiple pathways, highlighting MA1's potential as a promising therapeutic candidate.

## Graphical abstract

This study investigated the effects of the natural compound Malformin A1 (MA1) on the cytoskeleton that regulates cell proliferation and migration. Disruption of the cytoskeleton can impair these processes and promote cancer cell death. MA1 disrupted cytoskeletal organization, induced DNA damage, inflammation, activated autophagy, and pyroptosis, leading to cancer cell death. Created in biorender. Tamimi, Y. (2026) https://BioRender.com/c6rgjwf.

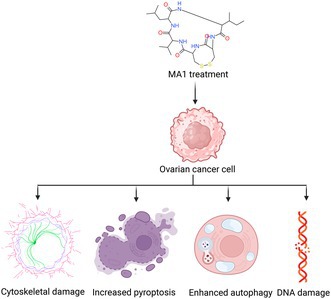

**Abbreviations**
CPT: camptothecin (CPT)
MA1: Malformin A1
MTA: microtubule‐targeting agents
OC: ovarian cancer
T.A2780CP: MA1‐treated A2780CP cells
T.A2780S: MA1‐treated A2780S cells

Ovarian cancer (OC) represents the eighth most common malignancy affecting women worldwide. The disease poses a clinical challenge because the majority of patients are diagnosed at advanced stages (III/IV). This creates a cascade of clinical complications, including inadequate debulking, initial response to chemotherapy, followed by drug resistance, and disease recurrence. These factors contribute to high mortality rates due to OC, underscoring the urgent need for improved early detection and novel therapeutic approaches [1, 2]. Consequently, there is a growing interest in alternative treatment modalities, with natural product‐derived compounds emerging as promising candidates. Due to reduced cytotoxicity and various protective biological effects, natural products are widely considered as substitutes for traditional cancer therapies [3]. Within this research landscape, strategies targeting the cytoskeletal network, specifically actin filaments and microtubules, have gained considerable attention as potential therapeutic approaches.

Cytoskeleton is a dynamic network composed of three distinct polymer structures: microtubules, actin filaments, and intermediate filaments. This complex framework maintains the structured integrity of intracellular components and orchestrates essential cellular functions. Microtubules, constructed from α‐and β‐tubulin subunits, polymerize to form protofilaments that serve multiple critical roles. These structures create an organizational network for intracellular protein and organelle trafficking, preserve cell morphology, and ensure accurate chromosome segregation during mitosis [4]. The therapeutic potential of natural compounds that target cytoskeletal components has gained significant attention in cancer research. These compounds demonstrate the ability to disrupt cytoskeletal networks, thereby affecting fundamental cellular processes and triggering cell death pathways. Chondramide, for example, induces apoptosis by specifically disrupting actin filaments [5]. Plant‐derived compound paucatalinone‐A has shown promise by enhancing mitochondrial damage and promoting cytoskeletal disruption, ultimately leading to apoptotic cell death in osteosarcoma [3]. Interestingly, a naturally occurring plant compound, Gaudichaudione‐H stimulates a unique form of cell death known as disulfidptosis by inducing abnormal disulfide bond formation between cytoskeletal proteins [6]. Similarly, withaferin‐A demonstrates anticancer properties by reducing β‐tubulin levels and arresting growth in human breast cancer cells [7].

Due to their crucial role in cell division, microtubules represent key therapeutic targets in tumor chemotherapy. Microtubule‐targeting agents (MTAs) bind to microtubules or tubulin monomers, disrupting microtubule organization and leading to mitotic arrest that induces cell death through intrinsic apoptosis pathways. Consequently, MTAs are widely employed in cancer treatment [8, 9]. Delgado et al. [8] previously demonstrated that distinct cell death pathways are activated by cytoskeletal disruption, with the specific pathway depending on the cell cycle phase during which microtubule depolymerization occurs. By disrupting the microtubule network, MTAs effectively suppress cell proliferation and promote cell death [10]. However, resistance mechanisms can limit therapeutic efficacy. In colon cancer, camptothecin (CPT) activates vimentin signaling pathways, which enhance cell survival to delay apoptosis [11].

Marine organisms represent a rich reservoir of novel bioactive metabolites with significant anticancer potential [12, 13, 14]. The field of marine drug discovery has expanded rapidly over the past decade, revealing remarkable cytotoxic properties. Previously, we screened 40 compounds and extracts derived from the Omani marine environment for anticancer activities. Among these, Malformin A1 (MA1) exhibited potent cytotoxic activity against the MCF7 breast cancer cell line [15]. MA1 is a cyclic pentapeptide biosynthesized by various marine fungi, including *Aspergillus niger* [16]. Its molecular structure was previously characterized by Koizumi Y. et al. [17]. MA1 exhibits diverse bioactive properties, including teratogenic, antibacterial, fibrinolytic, and cytotoxic activities [17]. This compound has shown a broad spectrum of cytotoxicity against multiple human cancer cell lines, including those derived from lung, pancreatic, breast, cervical, colorectal, and central nervous system [18]. In prostate cancer models, MA1 has been shown to induce cancer cell death through multiple mechanisms, including apoptosis, necrosis, and autophagy, primarily by promoting oxidative stress and causing mitochondrial damage [19].

This study aimed to elucidate the mechanisms by which MA1‐induced cytoskeletal impairment contributes to cell death in OC. Specifically, we investigated alterations in autophagy pathways, DNA damage responses, and pyroptosis markers to characterize MA1's cytotoxicity mechanisms beyond the conventional apoptotic pathways.

## Materials and methods

### MA1 procurement and preparation

Malformin A1 (MA1) was purchased from Boc Sciences (Shirley, NY, USA) and reconstituted as a 1 mg·mL^−1^ stock solution in dimethyl sulfoxide (DMSO; Sigma‐Aldrich, st. Louis, MO, USA). Stock solutions were stored at −20 °C until use.

### Cell culture and treatment

A2780S (RRID:CVCL_0134) and A2780CP (RRID:CVCL_0135) cells were kindly provided by Dr. Benjamin K. Tsang, University of Ottawa, Canada. A2780S and A2780CP cells were cultured in 25‐cm^2^ flasks and treated with MA1 at previously determined IC50 concentrations (0.23 μm for A2780S and 0.34 μm for A2780CP) for 24 h. Cell line identity was verified within the past 3 years using short tandem repeat (STR) profiling, with results confirmed against established reference profiles. All cells were routinely tested for mycoplasma contamination and returned negative results.

### Western blotting

For protein extraction, cells were lysed using RIPA lysis buffer (Thermo Fisher Scientific, Waltham, MA, USA), and total protein concentration was determined using the Bradford assay and quantified with a Nanodrop ND‐1000 spectrophotometer (Thermo Fisher Scientific). Equal amounts of protein (50–100 μg) were separated by SDS/PAGE on 10% polyacrylamide gels and transferred to PVDF or nitrocellulose membranes. Membranes were blocked with 5% bovine serum albumin (BSA) in Tris‐buffered saline with tween‐20 (TBS‐T) for 1 h at room temperature, followed by overnight incubation at 4 °C with primary antibodies. The following primary antibodies were used: (β‐actin: sc‐47 778, Caspase 1: sc‐392 736, c‐myc: sc‐42, Santa Cruz Biotechnology, Dallas, TX, USA); α‐tubulin (#2144), ATG5 (#2630), BAD (#9292), β‐tubulin (#15115), Cleaved caspase3 (#9661S), FADD (#2782S), GAPDH (#2118), LC3B (#2775), Phospho‐H2AX (#2577S), from Cell Signaling Technology (Danvers, MA, USA); BAX (#33‐6400), β‐catenin (#PA5‐77934), Caspase3 (#35‐1600Z), N‐cadherin (#PA5‐17526), TRADD (#PA5‐34807), Vimentin (#PA5‐27231), Thermo Fisher Scientific ((Waltham, MA, USA); Beclin (#ab210498), FAT4 (#ab130076) Abcam, Cambridge, UK). After washing with TBS‐T, membranes were incubated with appropriate HRP‐conjugated secondary antibodies (anti‐rabbit or anti‐mouse IgG, 1 : 5000 dilution) for 1 h at room temperature. Protein bands were visualized using Pierce ECL Western Blotting Substrate (Thermo Fisher Scientific) and detected with the iBright CL1000 imaging system (Invitrogen, CA, USA).

Because MA1 treatment caused degradation of commonly used housekeeping proteins (β‐actin and GAPDH), protein quantification was normalized to total protein content using Coomassie Brilliant Blue staining [20, 21]. For each sample, the intensity of the target protein band was normalized to the total protein density of its corresponding lane, as determined by Coomassie staining. This normalization approach effectively accounts for variations in sample loading and ensures more accurate quantification of protein expression levels.

### Total RNA extraction and cDNA synthesis

Total RNA was extracted from subconfluent A2780 CP and A2780 S cells using the PureLink RNA Mini Kit (Invitrogen, CA, USA) following the manufacturer's protocol. RNA concentration and purity were determined using a NanoDrop ND‐1000 spectrophotometer (Thermo Fisher Scientific). To eliminate potential genomic DNA contamination, RNA samples were treated with RNA‐free DNase (Qiagen, Hilden, Germany). RNA integrity was assessed by 1% agarose gel electrophoresis to confirm the presence of intact 28S and 18S ribosomal RNA bands. Subsequently, high‐quality RNA samples were reverse transcribed into complementary DNA (cDNA) using the High‐Capacity cDNA Reverse Transcription kit (Applied Biosystems, CA, USA).

### Quantitative real‐time PCR (qRT‐PCR) analysis

qRT‐PCR was conducted using optical 96‐well plates (Applied Biosystems) on an Applied Biosystems 7500 Fast Real‐Time PCR System. Each 20 μL reaction contained: 10 μL TaqMan Universal PCR Master Mix (Applied Biosystems), 1 μL cDNA template (25 ng total RNA), 8 μL RNase‐free water. The PCR amplification protocol consisted of an initial incubation at 50 °C for 2 min, followed by enzyme activation at 95 °C for 10 min, and 45 cycles of denaturation at 95 °C for 15 s and annealing/extension at 60 °C for 1 min. A no‐template control was included in each run.

### Immunofluorescent analysis procedure

A2780 S and A2780 CP cells were cultured on positively charged glass slides in DMEM/F12 medium supplemented with 10% FBS. Cells were rinsed with PBS (Gibco, Grand Island, New York, USA) and fixed in 70% ethanol for 15 min at room temperature. Following permeabilization with 0.1% Triton X‐100, cells were blocked with 5% goat serum for 30 min to prevent nonspecific binding. Cells were then incubated overnight at 4 °C with primary antibody at a 1 : 100 dilution. After washing, cells were exposed to fluorescent secondary antibody (Alexa Fluor; Cell Signaling, USA) for 1 h at room temperature. Cells were counter‐stained with Hoechst 33258 to visualize nuclei and mounted using glycerol/PBS medium. Fluorescence imaging was performed using a Nikon H600L and Olympus fluorescent microscope.

### Transmission electron microscopy (TEM) analysis

To investigate MA1‐induced cell death mechanisms, A2780 CP cells were treated with 0.34 μm MA1. Cells were then fixed, dehydrated through a graded ethanol series, and embedded in epoxy resin. Ultra‐thin sections (70–90 nm) were prepared using an ultramicrotome and stained with uranyl acetate and Reynold's lead citrate. TEM imaging was performed to visualize ultrastructural changes associated with cell death.

### Bioinformatic analysis

Pearson's correlation analysis was performed using the GEPIA database to explore the relationship between cytoskeletal gene expression and apoptotic markers (BCL2, BCL‐XL) as well as the autophagy marker (ATG5). Correlation coefficient and statistical significance were determined to assess gene expression relationships.

### Molecular docking studies

The target proteins and the ligand MA1 were retrieved and prepared using *in silico* tools for molecular docking analysis. The PDB files for Vimentin (PDB ID‐1GK4), β‐catenin (PDB ID‐1JDH), β‐tubulin (PDB ID‐1JFF), α‐tubulin (PDB ID‐5FNV), N‐cadherin (PDB ID‐1NCJ), GAPDH (PDB ID‐1ZNQ), Actin (PDB ID‐3BYH), AKT (PDB ID‐3QKM), STAT3 (PDB ID‐6NJS), YAP1 (PDB ID‐3KYS), and FAT4 (PDB ID‐8EGS) were retrieved from RSC Protein Data Bank (www.rcsb.org). The 3D structure for Malformin A1 was downloaded in SDF format from PubChem (https://pubchem.ncbi.nlm.nih.gov/). Molecular docking was performed using the Vina Wizard module in pyrx. Protein and ligand preparations were carried out with the autodock Auxiliary Tools (ADT) version 4.2, and AutoDock Vina was used for the docking simulations. Prior to docking, all water molecules were removed from the protein structure to expose the amino acid residues. Polar hydrogens were added, and Kollman charges were added to the amino residues. The processed protein structures were then saved in PDBQT format (Protein Data Bank (PDB), Partial Charge (Q), and Atom Type (T)). For ligand preparation, polar hydrogens and Gasteiger charges were added, while nonpolar hydrogens were merged to generate stable ligand conformation. The resulting ligand structures were also saved in PDBQT format. Following receptor and ligand preparation, grid maps were generated using adt version 4.2, with grid dimensions set to encompass the entire receptor binding region. The molecular interactions between Malformin A1 and the selected protein targets were visualized in three dimensions using the pymol Molecular Graphics System (Version 2.0.7) and in two dimensions using discovery studio visualizer (BIOVIA, Dassault Systèmes, 2023).

### Statistical analysis

Statistical significance was assessed using Student's *t*‐test, one‐way ANOVA, and Friedman test with ibm spss Version 23 and microsoft excel 2013. Significance levels were defined as follows: *P < 0.05, **P < 0.01, ***P < 0.001, and ****P < 0.0001. Data are presented as mean ± standard deviation (SD) from three independent experiments, each performed in triplicate.

## Results

### Expression of cytoskeleton‐related genes in ovarian cancer cells

**Fig. 1:**
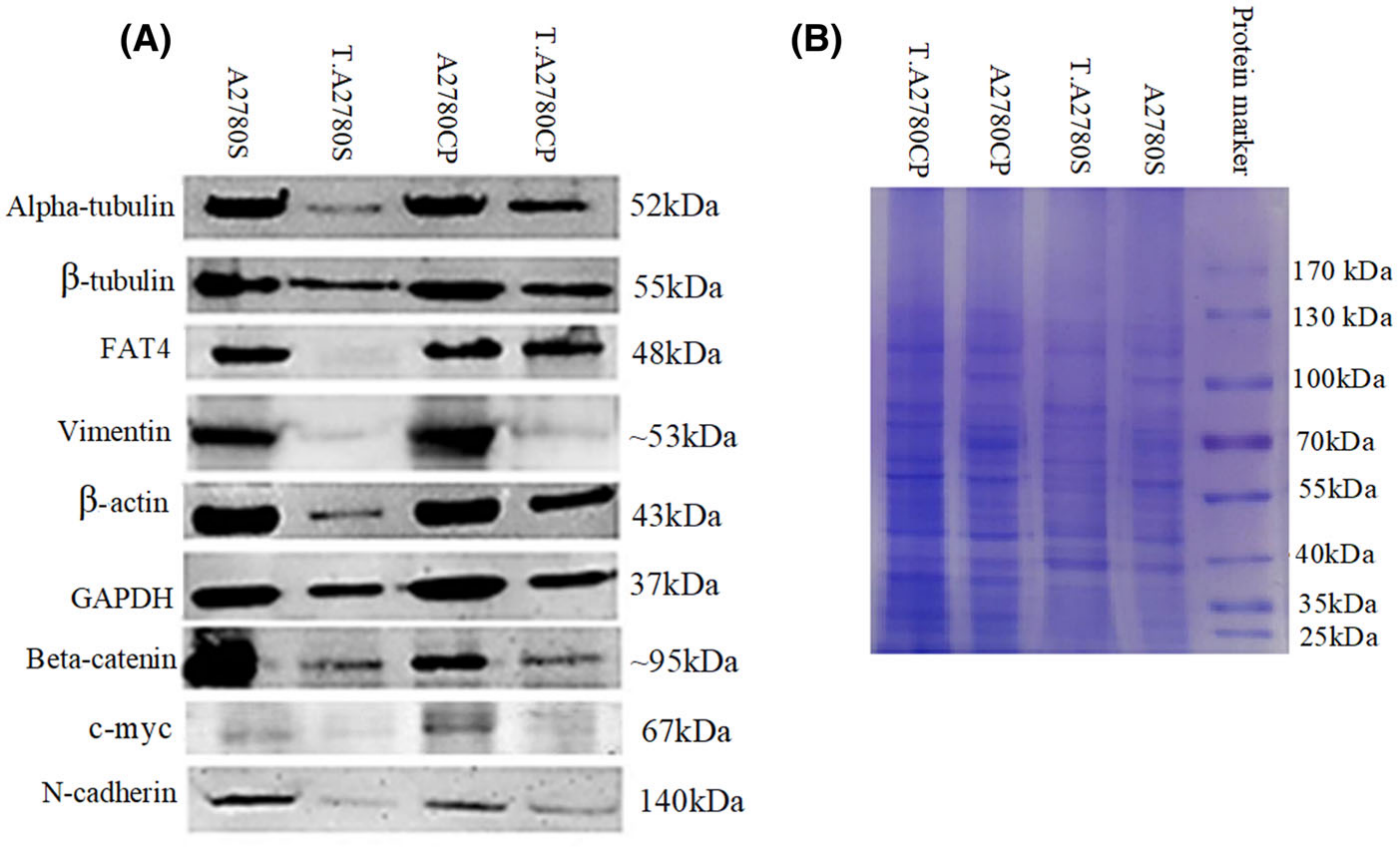
(A) Protein expression levels of α‐Tubulin, β‐Tubulin, FAT4, Vimentin, β‐Actin, GAPDH, Beta‐catenin, c‐Myc, and N‐cadherin were assessed using western blot analysis after 24 h of MA1 treatment in ovarian cancer cells A2780S and A2780CP; (B) The protein bands were normalized to the total protein load using Coomassie blue staining. Full‐length blots are included in Fig. S1. Normalized protein band intensity using imagej is included in Fig. S2.

**Fig. 2:**
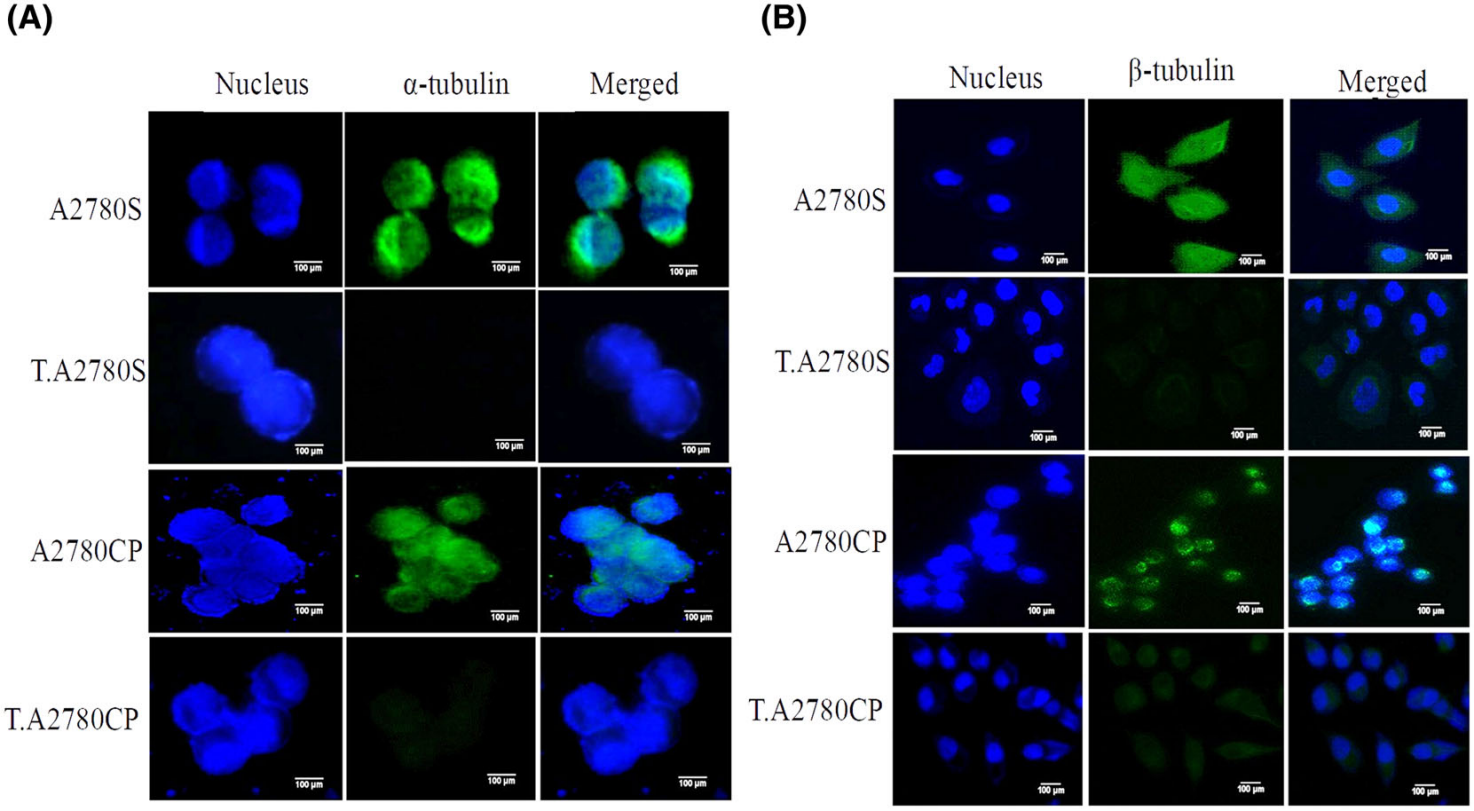
Immunofluorescence staining of healthy cells compared to MA1‐treated cells for 24 h is shown for (A) α‐tubulin; (B) β‐Tubulin. Nucleus staining was performed using Hoechst stain, as shown in the left column. The middle column displays the specific protein being tested, while the right column represents a merge of the two columns. All images were captured at 40× magnification. The quantitative analysis of fluorescence intensity using imagej is included in Tables S1, S2. MA1 treated A2780S cells: T.A2780S; MA1 treated A2780CP cells: T.A2780CP. Scale bars = 100 μm for all panels.

**Fig. 3:**
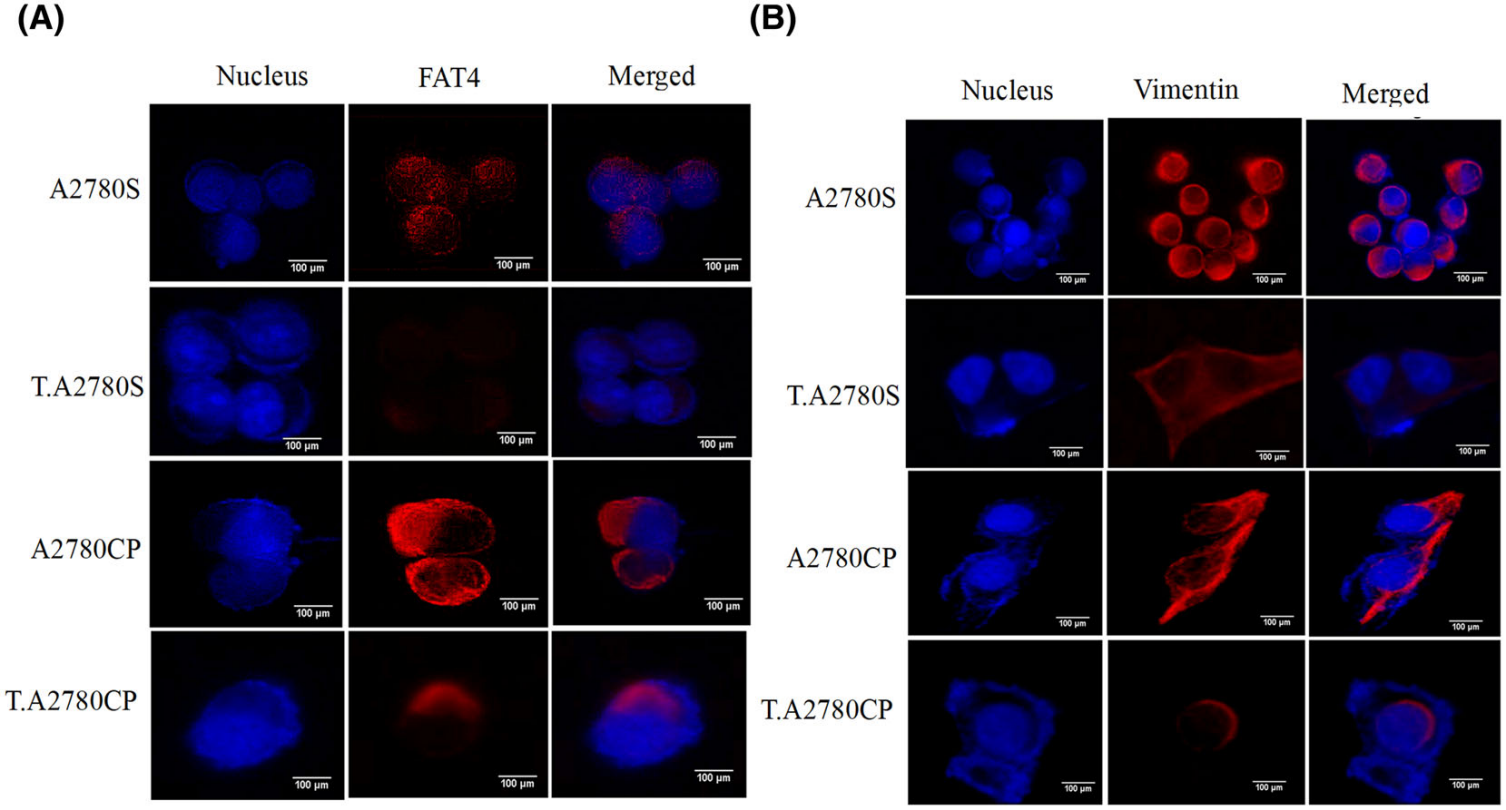
Immunofluorescence staining of healthy cells compared to MA1‐treated cells for 24 h is shown for (A) FAT4; (B) Vimentin. Nucleus staining was performed using Hoechst stain, as shown in the left column. The middle column displays the specific protein being tested, while the right column represents a merge of the two columns. All images were captured at 40× magnification. The quantitative analysis of fluorescence intensity using imagej is included in Tables S1, S2. MA1 treated A2780S cells: T.A2780S; MA1 treated A2780CP cells: T.A2780CP. Scale bars = 100 μm for all panels.

**Fig. 4:**
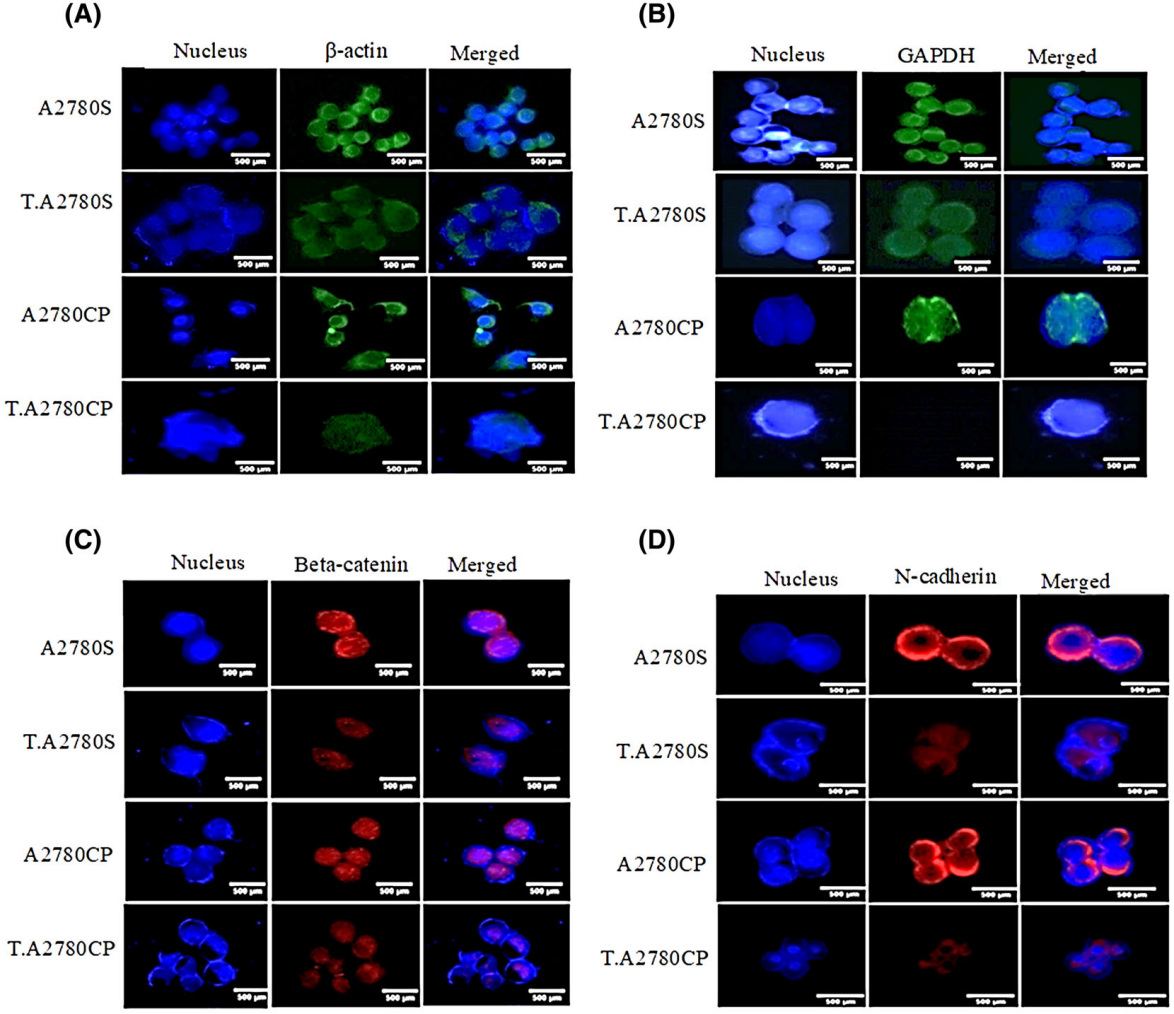
Immunofluorescence staining of (A) β‐Actin; (B) GAPDH; (C) β‐catenin; and (D) N‐cadherin in healthy cells compared to cells treated with MA1 for 24 h. Nucleus staining was performed using Hoechst stain, as shown in the left column. The middle column displays the specific protein being tested, while the right column represents a merge of the two columns. All images were captured at 40× magnification. The quantitative analysis of fluorescence intensity using imagej is included in Tables S1, S2. MA1 Treated A2780S cells: T.A2780S; MA1 treated A2780CP cells: T.A2780CP. Scale bars = 500 μm for all panels.

To investigate the influence of MA1 on cellular morphology and structural organization, we evaluated the expression of key cytoskeleton and associated proteins, including α‐tubulin, β‐tubulin, FAT4, vimentin, β‐actin, GAPDH, β‐catenin, c‐Myc, and N‐cadherin in ovarian cancer cells using western blotting and immunofluorescence analysis (Figs 1, 2, 3, 4). Western blot analysis revealed varying degrees of downregulation following MA1 treatment in both A2780S and A2780CP cells (Fig. 1A, Full‐length western blots and normalized band intensities are shown in Figs S1 and S2). Moreover, MA1 treatment was associated with reduced expression of β‐catenin and c‐Myc, indicating decreased levels of key components of the Wnt/β‐catenin signaling pathway. Since MA1 treatment affected the expression of housekeeping genes such as actin and GAPDH, protein bands were normalized against total protein loading to ensure accurate quantification (Fig. 1B).

Immunofluorescence analysis supported these findings, confirming reduced fluorescence intensity in MA1‐treated cells compared with untreated controls (Figs 2, 3, 4, Quantitative analysis of fluorescence intensity is provided in Tables S1 and S2). Among the tubulin proteins, α‐tubulin showed a more pronounced decrease in treated A2780S cells relative to A2780CP cells. This differential response was particularly evident at the cellular level through immunofluorescence microscopy, where treated cells displayed markedly diminished fluorescence signals (Fig. 2). Similarly, FAT4, vimentin, and β‐actin showed decreased protein expression following treatment, with more substantial reductions observed in the sensitive A2780S cell line. Immunofluorescence staining of these proteins revealed consistent patterns of downregulation post‐treatment (Figs 3, 4A). MA1 treatment also significantly affected GAPDH and β‐catenin expression, with notable decreases observed in both A2780S and A2780CP cell lines (Fig. 4B,C).

**Fig. 5:**
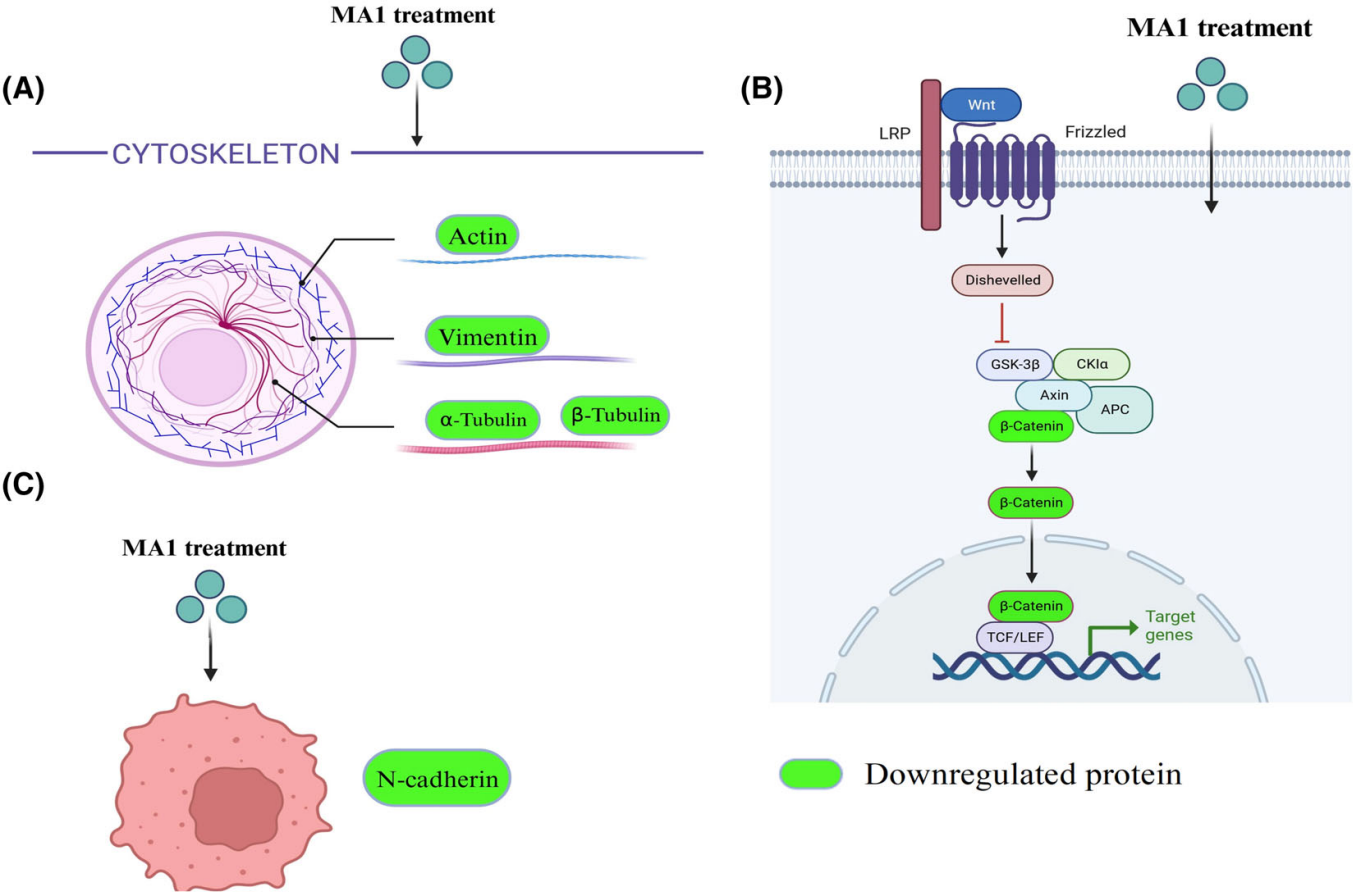
Schematic representation of cytoskeleton network disruption following MA1 treatment. Downregulated proteins are indicated by green boxes (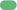). Created in biorender. Tamimi, Y. (2026) https://BioRender.com/xjovews.

N‐cadherin displayed a similar degradation pattern, which was more pronounced in treated A2780CP cells, particularly evident in single‐cell analyses (Fig. 4D). The reduced fluorescence intensity following MA1 treatment suggests both transcriptional downregulation and enhanced degradation of cytoskeletal proteins. Consistently, qRT‐PCR analysis revealed decreased mRNA expression levels of these proteins, indicating transcriptional and/or post‐transcriptional regulation by MA1. In parallel, western blot analysis showed reduced protein abundance, supporting the occurrence of cytoskeletal protein degradation. Collectively, these results indicate that MA1 disrupts cytoskeletal integrity by inhibiting gene expression and promoting the degradation of structural proteins. A schematic overview of the molecular alterations observed in the cytoskeletal network following MA1 treatment is presented in Fig. 5, with downregulated proteins indicated in green boxes.

### Expression of apoptosis‐related genes in ovarian cancer cells

**Fig. 6:**
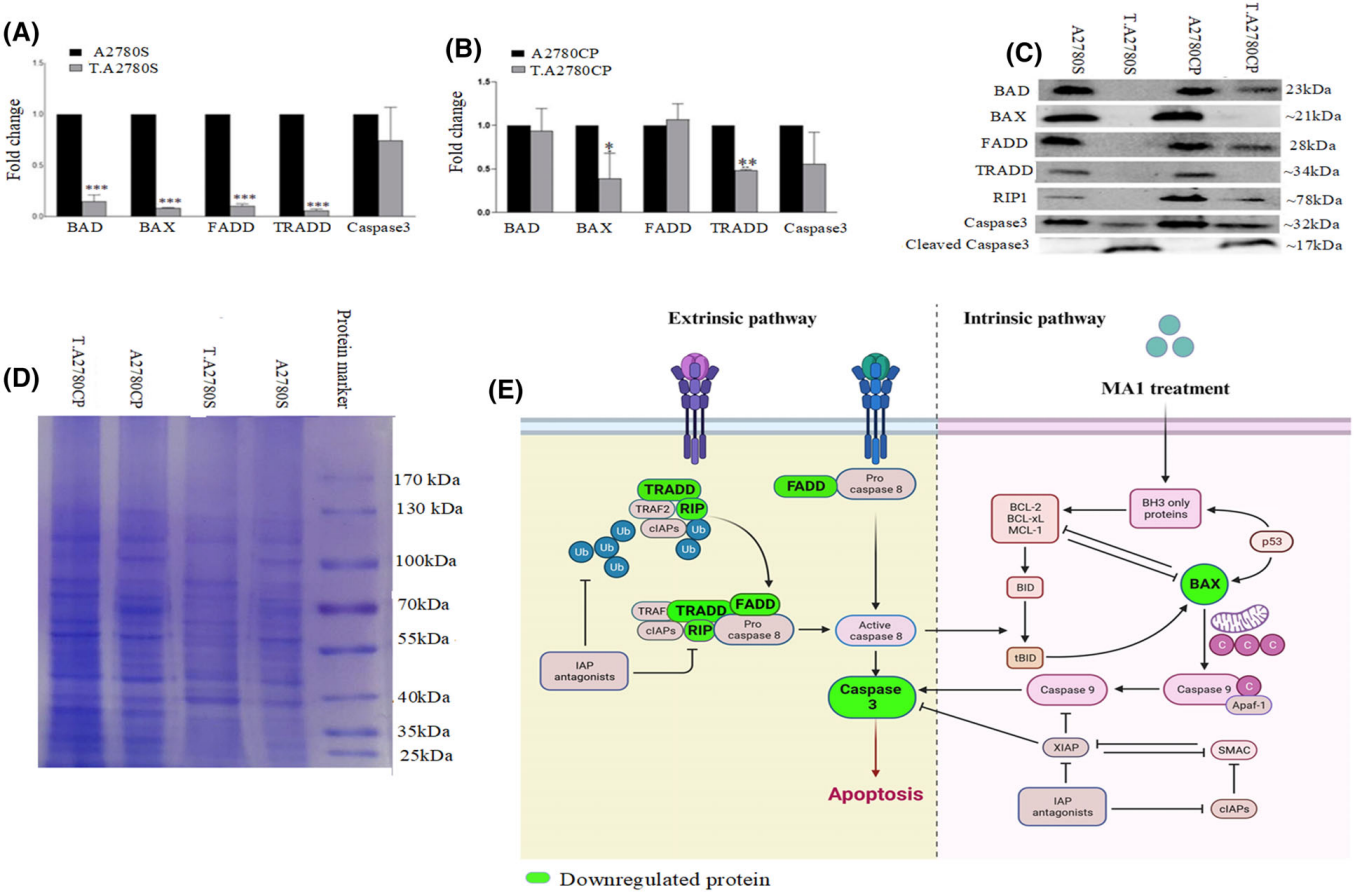
qRT‐PCR analysis of BAD, BAX, FADD, TRADD, and Caspase3 gene expression levels in: (A) A2780S cells, and; (B) A2780CP cells following MA1 treatment. The gene expressions were normalized to PUM1; Data represent the mean ± SD of three independent experiments. Statistical significance is denoted as *P < 0.05, **P < 0.01, ***P < 0.001; (C) Western blot analysis showing protein expression levels of BAD, BAX, FADD, TRADD, RIP1, Caspase3, and Cleaved caspase3 in A2780S and A2780CP ovarian cancer cells after 24 h of MA1 treatment. Full‐length blots are included in Fig. S3. Normalized protein band intensity using imagej is included in Fig. S4. (D) Protein bands were normalized to the total protein load using Coomassie blue staining for A2780S and A2780CP cells. (E) Schematic overview of molecular alterations associated with apoptosis following MA1 treatment. Proteins downregulated upon MA1 treatment are represented by green boxes (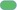). Created in biorender. Tamimi, Y. (2026) https://BioRender.com/13mrf3u. MA1 treated A2780S cells: T.A2780S; MA1 treated A2780CP cells: T.A2780CP.

**Fig. 7:**
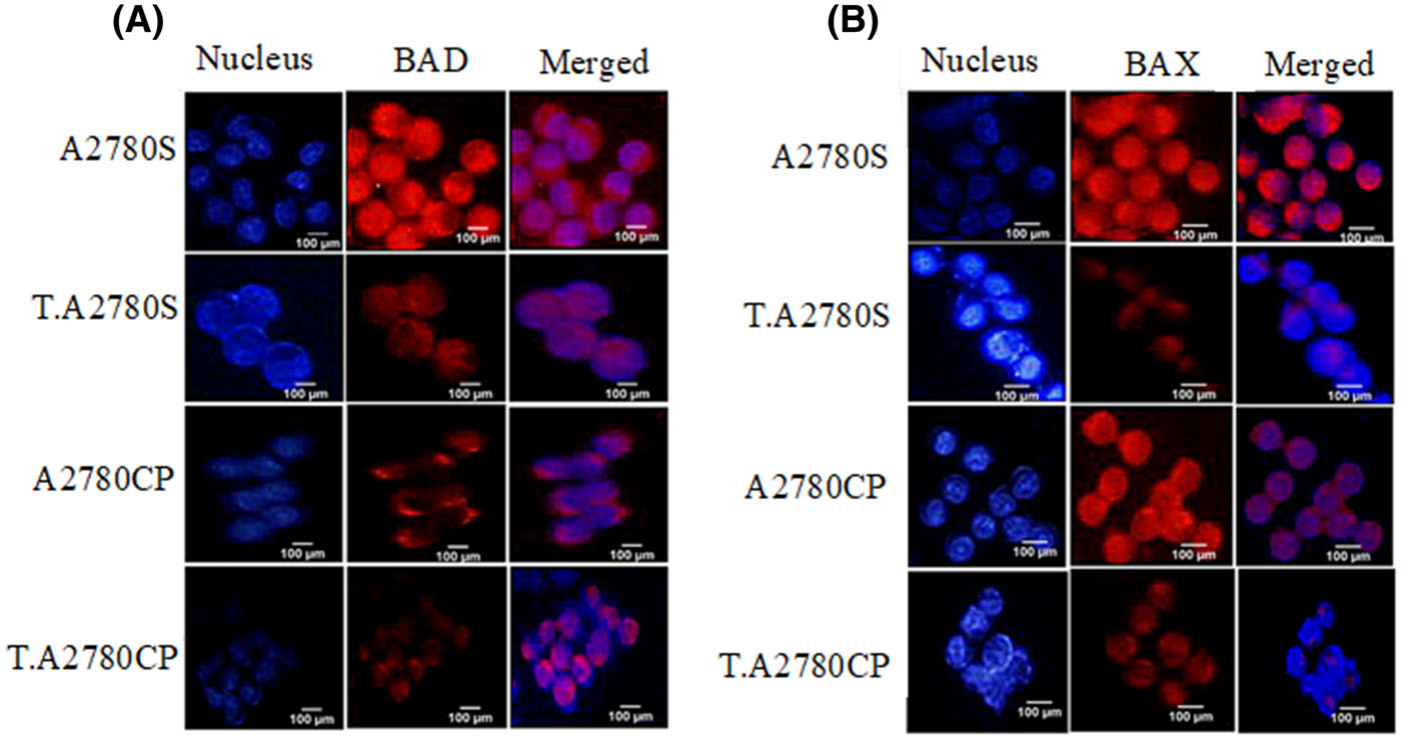
Immunofluorescence staining of: (A) BAD; (B) BAX proteins after 24 h of MA1 treatment. Nucleus staining was performed using Hoechst stain, shown in the left column, while the right column represents the merge of the two columns. All images were captured at a magnification of 40×. The quantitative analysis of fluorescence intensity using imagej is included in Table S3. MA1 treated A2780S cells: T.A2780S; MA1 treated A2780CP cells: T.A2780CP. Scale bars = 100 μm for all panels.

**Fig. 8:**
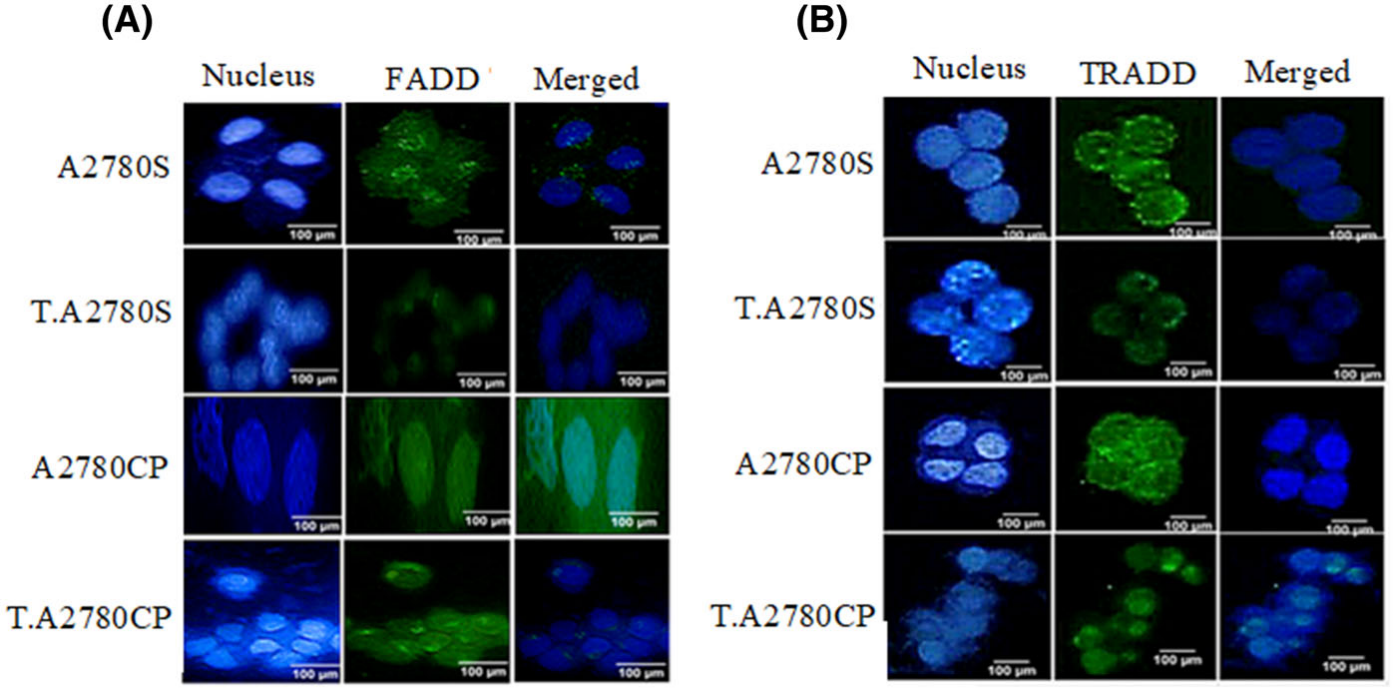
Immunofluorescence staining of: (A) FADD; (B) TRADD proteins after 24 h of MA1 treatment. Nucleus staining was performed using Hoechst stain, shown in the left column, while the right column represents the merge of the two columns. All images were captured at a magnification of 40×. The quantitative analysis of fluorescence intensity using imagej is included in Table S3. MA1 treated A2780S cells: T.A2780S; MA1 treated A2780CP cells: T.A2780CP. Scale bars = 100 μm for all panels.

**Fig. 9:**
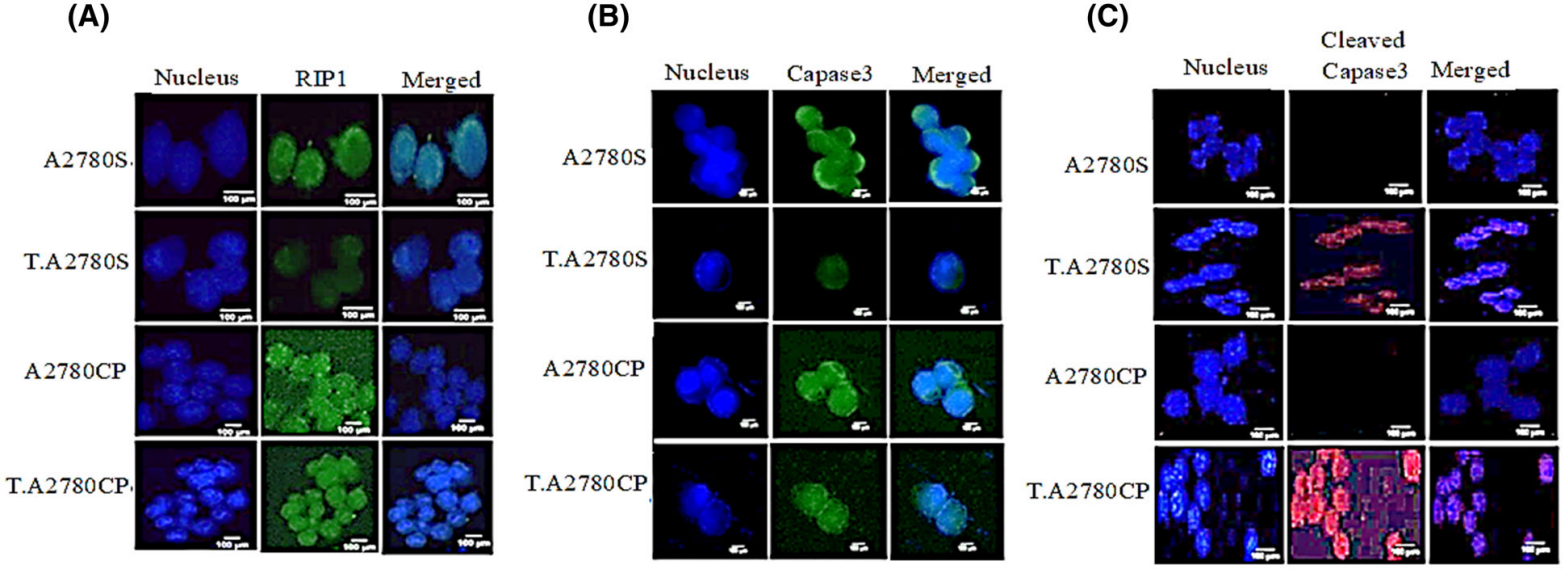
Immunofluorescence staining of: (A) RIP; (B) Caspase3, and; (C) Cleaved Caspase3; proteins after 24 h of MA1 treatment. Nucleus staining was performed using Hoechst stain, shown in the left column, while the right column represents the merge of the two columns. All images were captured at a magnification of 40×. The quantitative analysis of fluorescence intensity using imagej is included in Table S3. MA1 treated A2780S cells: T.A2780S; MA1 treated A2780CP cells: T.A2780CP. Scale bars = 100 μm for all panels.

To investigate the effect of MA1‐induced cytoskeletal disruption on apoptosis, we examined the expression levels of key apoptotic markers, including BAD, BAX, FADD, TRADD, RIP1, Caspase3, and Cleaved caspase3, using qRT‐PCR, western blotting, and immunofluorescence techniques (Figs 6, 7, 8, 9). qRT‐PCR analysis revealed differential responses between cell lines following MA1 treatment. In A2780S cells (T.A2780S), significant downregulation was observed for BAD, BAX, FADD, and TRADD. In contrast, A2780CP cells (T.A2780CP) showed a significant reduction only in BAX and TRADD expression levels (Fig. 6A,B).

Western blot analysis confirmed these gene expression findings at the protein level. MA1‐treated sensitive cells exhibited a significant decrease in multiple pro‐apoptotic markers (BAD, BAX, FADD, TRADD, Caspase3, and RIP1), while resistant A2780CP cells demonstrated significant reductions only in BAX, TRADD, and RIP1 protein levels compared to untreated controls (Fig. 6C, Full‐length western blots and normalized band intensities are shown in Figs S3, S4). Due to MA1‐induced degradation of housekeeping genes such as actin and GAPDH, protein bands were normalized against total protein loading to ensure accurate quantification (Fig. 6D). Immunofluorescence analysis corroborated the western blot findings, demonstrating decreased fluorescence intensity in MA1‐treated cells compared to controls (Figs 7, 8, 9, Quantitative analysis of fluorescence intensity is provided in Table S3). The consistent differential expression of apoptotic markers at both the mRNA and protein levels suggests that MA1 modulates apoptotic pathways through coordinated transcriptional and translational regulatory mechanisms. These results collectively indicate that MA1 treatment leads to downregulation of pro‐apoptotic proteins, with more extensive effects observed in the sensitive A2780S cell line. A schematic representation of molecular changes observed within the canonical apoptotic pathway following MA1 treatment is presented in Fig. 6E, with downregulated proteins highlighted in green boxes.

### Expression of autophagy‐related genes in ovarian cancer cells

**Fig. 10:**
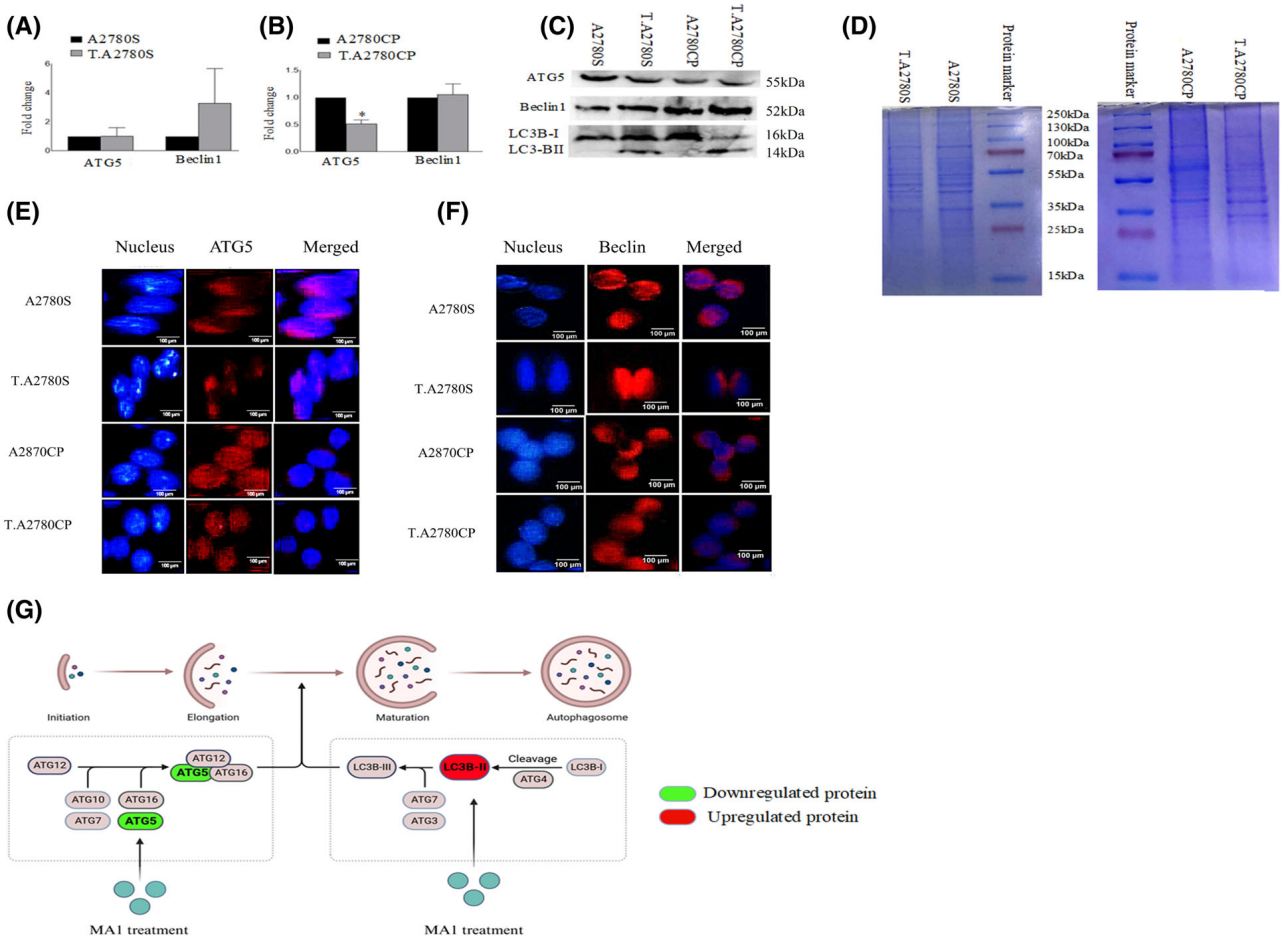
qRT‐PCR analysis of ATG5 and Beclin 1 gene expression levels in (A) A2780S cells, and; (B) A2780CP cells. The gene expressions were normalized to PUM1; Data represent the mean ± SD of three independent experiments. Statistical significance is denoted as *P < 0.05. (C) Western blot analysis displaying the expression of ATG5, Beclin 1, LC3B‐I, and LC3‐BII, proteins in ovarian cancer cells 24 h after MA1 treatment; Full‐length blots are included in Fig. S5. Normalized protein band intensity using imagej is included in Fig. S6. (D) The protein bands were normalized to the total protein load using Coomassie blue staining; Immunofluorescence staining of (E) ATG5 and; (F) Beclin 1 proteins in cells treated with MA1 for 24 h. Nucleus staining was performed using Hoechst stain, shown in the left column, while the right column represents the merge of the two columns. All images were captured at a magnification of 40×. The quantitative analysis of fluorescence intensity using imagej is included in Table S3. Scale bars = 100 μm for all panels. (G) Schematic overview of molecular alterations associated with autophagy following MA1 treatment. Downregulated proteins are represented by green boxes (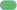), while upregulated proteins are represented by red boxes (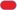). Created in biorender. Tamimi, Y. (2026) https://BioRender.com/98pyc0r. MA1 treated A2780S cells: T.A2780S; MA1 treated A2780CP cells: T.A2780CP.

To investigate the impact of MA1‐induced cytoskeletal disruption on autophagy, we examined the expression of key autophagy‐related markers, specifically ATG5, Beclin 1, and LC3BI/II, using qRT‐PCR, western blotting, and immunofluorescence techniques (Fig. 10). Due to MA1‐induced degradation of housekeeping genes (actin and GAPDH), all protein bands were normalized against total protein loading to ensure accurate quantification.

qRT‐PCR analysis revealed differential autophagy gene expression responses between cell lines. ATG5 expression remained unchanged in MA1‐treated A2780S cells but was significantly downregulated in A2780CP cells (Fig. 10A,B). Beclin1 expression showed no significant change in MA1‐treated A2780S and A2780CP cells compared to untreated controls.

Western blot analysis demonstrated enhanced conversion of LC3BI to LC3BII in MA1‐treated cells, indicating increased autophagy flux (Fig. 10C,D, Full‐length western blots and normalized band intensities are shown in Figs S5, S6). This LC3B conversion pattern was consistent across both cell lines, further supporting autophagy activation following MA1 treatment. Immunofluorescence analysis revealed decreased tendency of ATG5 protein expression in both MA1‐treated A2780S and A2780CP cells (Fig. 10E, Quantitative analysis of fluorescence intensity is provided in Table S3), which correlated with the qRT‐PCR findings in A2780CP cells but contrasted with the unchanged mRNA levels observed in A2780S cells. This discrepancy suggests potential post‐transcriptional regulation of ATG5 in the sensitive cell line. These findings collectively indicate that MA1 treatment modulates autophagy through multiple mechanisms, with distinct responses observed between sensitive and resistant ovarian cancer cell lines. Furthermore, a tendency toward increased beclin‐1 levels was observed (Fig. 10F). A schematic overview of the molecular changes mapped onto the canonical autophagy pathway following MA1 treatment is presented in Fig. 10G, with downregulated proteins indicated in green boxes and upregulated proteins in red boxes.

### Ultrastructural analysis of MA1‐treated A2780CP cells

**Fig. 11:**
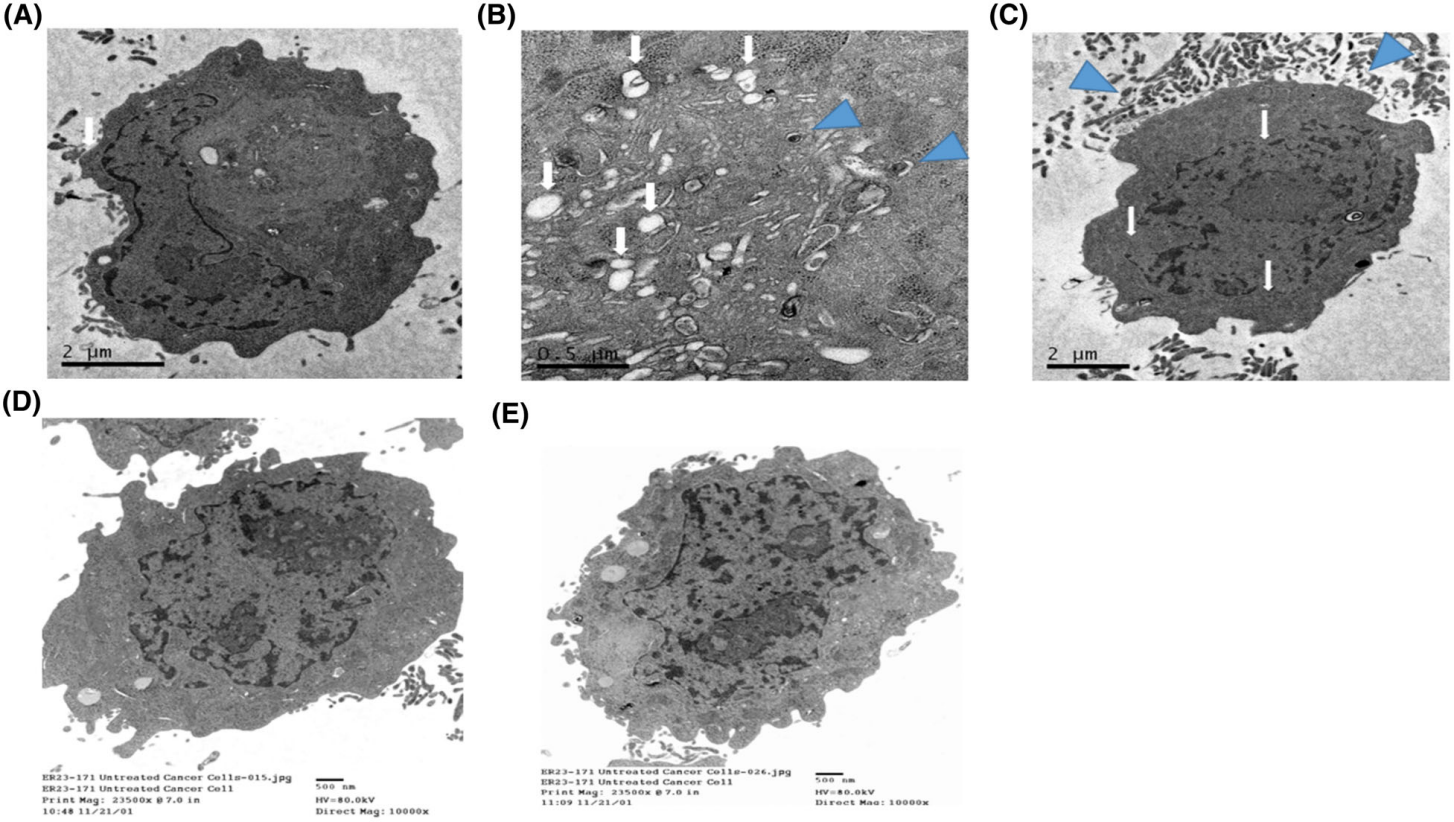
Transmission electron microscopy ultrastructural analysis of A2780CP cells treated with MA1 for 24 h: (A) At 15 000X magnification, apoptotic bodies (white arrow) indicating cell death are observed in MA1‐treated A2780CP cells; (B) At 50 000X magnification, an abundance of autovacuolization (white arrow) and autophagocytosis engulfing damaged proteins (arrowhead) is observed in MA1‐treated A2780CP cells; (C) At 12 000X magnification, chromatin segregation and early signs of nuclear membrane disruption (white arrow) are noticed in MA1‐treated A2780CP cells. Additionally, apoptotic bodies (arrowhead) are observed; (D, E) Untreated A2780CP cells show intact cellular membranes with no appearance of autophagosomes. Normal large nuclei with preserved cytoplasm are clearly identified at 10 000X magnification.

Transmission electron microscopy (EM) was used to examine the ultrastructural changes in MA1‐treated A2780CP cells. The analysis revealed multiple hallmarks of programmed cell death, including the formation of apoptotic bodies and the development of autophagic vacuoles containing cellular debris. Notably, numerous autophagosomes were observed encapsulating damaged organelles and proteins, indicating active autophagosome formation and protein degradation. Nuclear alterations were prominent, characterized by chromatin condensation and nuclear membrane fragmentation. These ultrastructural features collectively confirm the concurrent activation of both apoptotic and autophagic pathways in MA1‐treated A2780CP cells (Fig. 11).

### Detection of DNA damage marker ᵧ‐H2AX in MA1‐treated cells

**Fig. 12:**
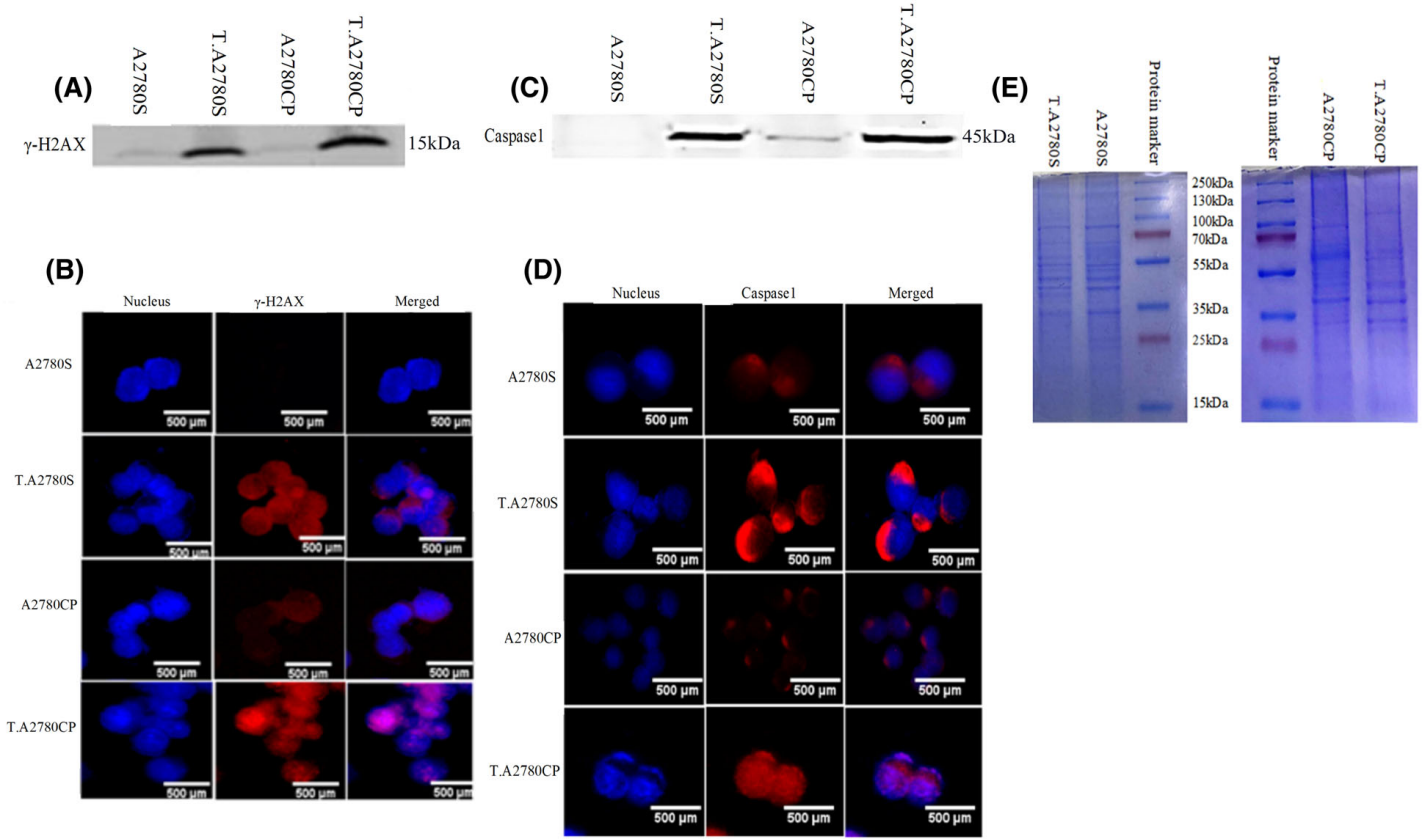
(A) Western blot analysis displaying the expression of γ‐H2AX protein in MA1‐treated ovarian cancer cells A2780S and A2780CP after 24 h of treatment. Protein bands were normalized to the total protein load using Coomassie blue staining. Full‐length blots are included in Fig. S7. Normalized protein band intensity using imagej is included in Fig. S8. (B) Immunofluorescence staining of γ‐H2AX in untreated and MA1‐treated cells for 24 h. All images were captured at a magnification of 40X. The quantitative analysis of fluorescence intensity using imagej is included in Table S3. Scale bars = 500 μm for all panels. (C) Western blot analysis displaying the expression of Caspase‐1 protein in ovarian cancer cells 24 h after MA1 treatment. Full‐length blots are included in Fig. S9. Normalized protein band intensity using imagej is included in Fig. S10. (D) Immunofluorescence staining of Caspase‐1 in untreated and MA1‐treated cells for 24 h. All images were captured at a magnification of 40X. Scale bars = 500 μm for all panels. The quantitative analysis of fluorescence intensity using imagej is included in Table S3. (E) Protein bands were normalized to the total protein load using Coomassie blue staining. MA1 treated A2780S cells: T.A2780S; MA1 treated A2780CP cells: T.A2780CP.

To assess DNA damage induced by MA1 treatment, we analyzed the expression of γ‐H2AX, a well‐established marker of DNA double‐strand breaks, using western blotting and immunofluorescence microscopy. Western blot analysis showed significantly elevated γ‐H2AX in MA1‐treated cells compared to untreated controls, indicating substantial DNA damage (Fig. 12A, Full‐length western blots and normalized band intensities are shown in Figs S7, S8). Immunofluorescence microscopy corroborated these findings, revealing increased γ‐H2AX in treated cells, which represents the cellular response to DNA double‐strand breaks (Fig. 12B, Quantitative analysis of fluorescence intensity is provided in Table S3). These results provide evidence that MA1 treatment induces significant DNA damage in ovarian cancer cells, contributing to the observed cytotoxic effects and activation of cell death pathways.

### Exploration of MA1‐induced alternative cell death pathways

To determine whether cytoskeletal disruption caused by MA1 triggers cell death through nonapoptotic mechanisms, we specifically examined pyroptosis as an alternative pathway. Using western blotting and fluorescent microscopy, we analyzed caspase‐1 expression levels in MA1‐treated A2780S and A2780CP cell lines. Results revealed elevated caspase‐1 levels in both cell lines following MA1 treatment, suggesting that MA1 induces pyroptotic cell death (Fig. 12C,D, Full‐length western blots and normalized band intensities are shown in Figs S9, S10; quantitative analysis of fluorescence intensity is provided in Table S3).

### Bioinformatic analysis

**Fig. 13:**
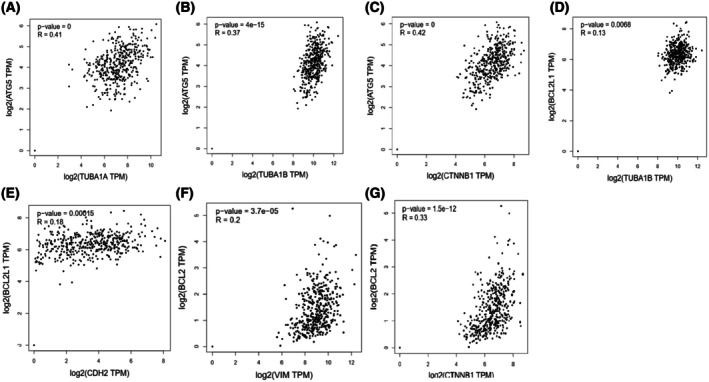
Pearson's correlation analysis between ATG5 and the following proteins: (A) *α*‐Tubulin; (B) *β*‐Tubulin; and (C) β‐catenin. Pearson's correlation analysis between BCL‐XL and the following proteins: (D) *β*‐Tubulin; (E) N‐cadherin. Pearson's correlation analysis between BCL2 and the following proteins: (F) Vimentin; and (G) β‐catenin.

Given our previous findings that MA1‐induced cytoskeletal disruption triggers cell death in A2780S and A2780CP cells and reduces cytoskeletal gene expression, we conducted Pearson's correlation analysis to examine the relationship between cytoskeletal gene expression and apoptotic/autophagy markers. Correlation analysis revealed several significant associations. The autophagy marker ATG5 showed positive correlations with multiple cytoskeletal components: α‐tubulin (*r* = 0.41, *P* < 0.05), β‐tubulin (*r* = 0.37, *P* = 4 × 10^−15^), and β‐catenin (*r* = 0.42, *P* < 0.05). Furthermore, anti‐apoptotic markers showed positive correlations with cytoskeletal proteins. BCL‐XL correlated with β‐tubulin (*r* = 0.13, *P* = 0.0068) and N‐cadherin (*r* = 0.18, *P* = 0.00015), while BCL2 displayed correlations with Vimentin (*r* = 0.20, *P* = 3.7 × 10^−5^), *R* = 0.2, and β‐catenin (*r* = 0.33, *P* = 1.5 × 10^−12^) (Fig. 13).

### Interaction of MA1 with selected target proteins

**Fig. 14:**
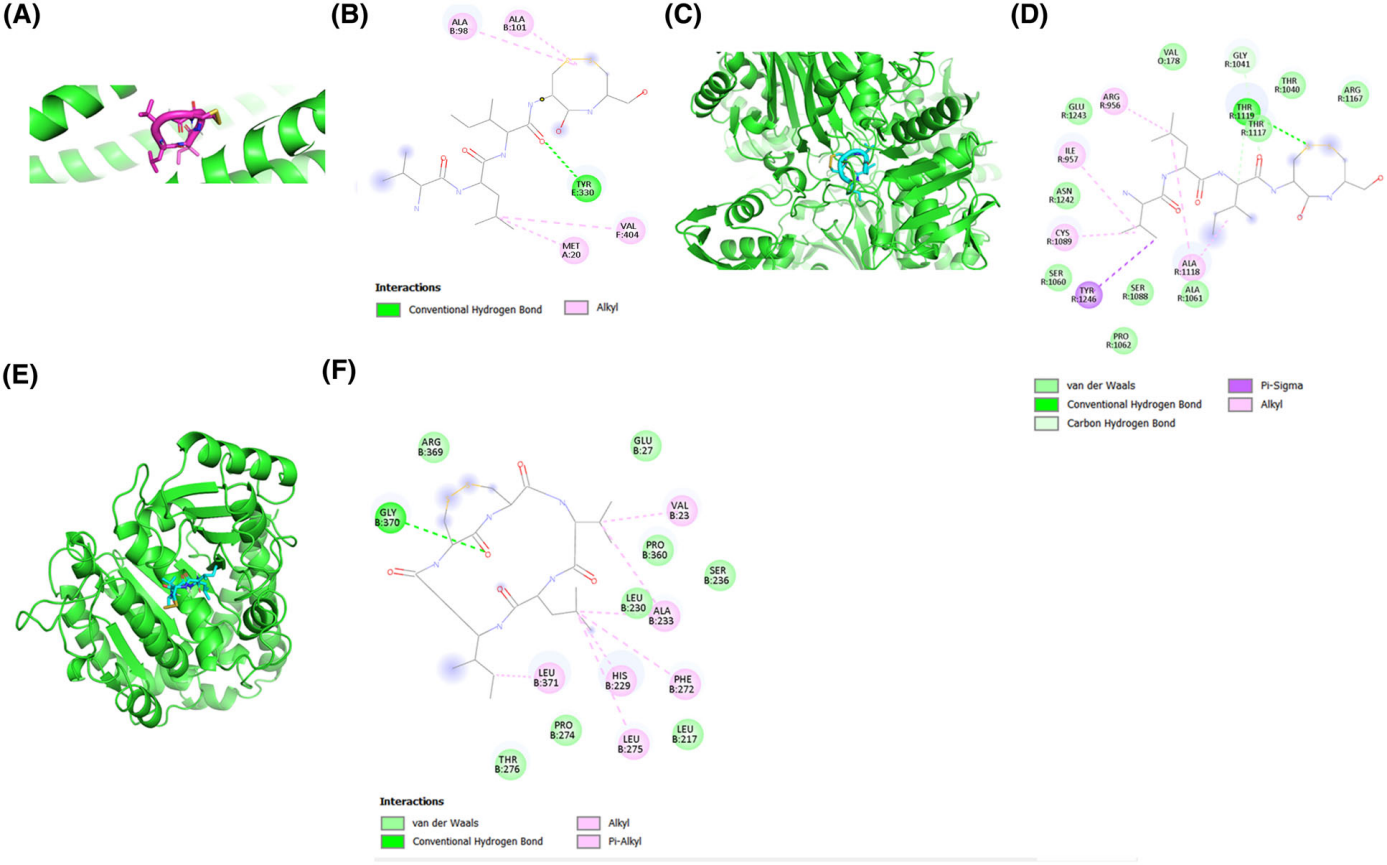
Molecular docking analysis of MA1 with Vimentin, GAPDH, and β‐tubulin proteins. The figure illustrates the three‐dimensional binding conformations and two‐dimensional interaction of MA1 with (A, B) Vimentin, (C, D) GAPDH, and (E, F) β‐tubulin, respectively. The 3D protein structures were obtained from the RCSB Protein Data Bank.

Molecular docking analysis was conducted to elucidate the mechanistic basis of MA1's anticancer potential by characterizing its interactions with key regulatory proteins involved in cytoskeletal dynamics (vimentin, actin, α‐tubulin, β‐tubulin, β‐catenin, N‐cadherin), metabolism (GAPDH), and cell survival signaling pathways (AKT, YAP1, STAT3, FAT4). The molecular docking analysis elucidated the interactions between MA1 and the selected target proteins (Fig. 14). Interestingly, MA1 showed significantly stable binding affinities, exceeding 6.5 kcal·mol^−1^ across all targets (Table 1). Among these, vimentin exhibited the strongest binding affinity of −8.1 kcal·mol^−1^. This interaction involved a key hydrogen bond at TYR330, along with multiple alkyl interactions with surrounding amino acids. A similar binding affinity (−8.1 kcal·mol^−1^) was observed with GAPDH, where hydrogen bonds were formed at Thr‐1117 and Thr‐1119, accompanied by π–π sigma interaction and other hydrophobic interactions that further stabilized the complex. MA1 also bound strongly to α‐tubulin and β‐tubulin, each with a binding affinity of −8.1 kcal·mol^−1^. The α‐tubulin interaction featured a hydrogen bond at Thr 276, whereas β‐tubulin formed a hydrogen bond at Gly370.

**Table 1:** Molecular docking binding scores of MA1 with selected target proteins.

Target proteins	Binding scores
Vimentin	−8.1
Beta‐catenin	−6.7
Beta‐tubulin	−8.1
N‐cadherin	−6.7
GAPDH	−8.1
Actin	−7.6
AKT	−7.7
STAT3	−6.6
YAP1	−6.7
FAT4	−7.7
Alpha tubulin	−7.5

For AKT and FAT4, MA1 exhibited binding affinities of −7.7 kcal·mol^−1^. In AKT, strong hydrogen bonds were identified at Asp292, Lys276, and Thr160. Notably, MA1 interacted directly with the AKT active site residue ASP292, suggesting a mechanism of competitive inhibition [22]. In the case of FAT4, MA1 formed interface hydrogen bonds at Asn249, supported by hydrophobic and van der Waals interactions that reinforced the binding stability.

The binding affinity of MA1 with actin was −7.6 kcal·mol^−1^, forming hydrogen bonds at Lys103 and Arg106, in addition to significant van der Waals and alkyl interactions.

MA1 displayed moderate binding affinities with YAP1 and N‐cadherin (−6.7 kcal·mol^−1^ each). Collectively, these findings highlight the robust interaction of MA1 with multiple cytoskeletal and signaling proteins, supporting its potential role in inducing cytoskeletal disruption and promoting cancer cell death.

## Discussion

We previously investigated MA1's effects on both normal ovarian cells (HOSE 6‐3) and OC cell lines, specifically comparing responses between cisplatin‐sensitive (A2780S) and cisplatin‐resistant (A2780CP) cells. Our previous results demonstrated the remarkable cytotoxic efficacy of MA1, even at submicromolar concentrations, with enhanced potency when combined with cisplatin [18]. Further molecular analysis revealed that MA1‐induced cytotoxicity resulted in significant downregulation of *bcl2* and *p53* gene expression. However, we observed no significant alterations in *caspase 3* or *caspase 9* expression levels, suggesting that apoptosis may not be the primary mechanism underlying MA1‐induced cytotoxicity in OC cell lines [18]. Building on these findings, this study investigated the cytoskeletal destabilization mechanisms induced by MA1 and the subsequent pathways leading to cell death in OC. Our findings demonstrate that MA1 treatment causes profound disruption of the cytoskeletal network in both A2780S and A2780CP cells, triggering multiple interconnected cell death pathways. MA1 treatment significantly reduced α‐tubulin and β‐tubulin expression, indicating potent inhibition of microtubule formation.

This mechanism of cytotoxicity makes MA1 among microtubule‐targeting agents (MTAs), which have demonstrated considerable efficacy across various cancer types [23]. Natural compounds with MTA‐like properties, such as MA1, disrupt microtubule dynamics essential for cellular transport and division, providing a mechanistic basis for their anticancer activity [24].

The observed reduction in β‐actin expression, particularly in the platinum‐sensitive cell line (A2780s), further emphasizes the role of MA1's comprehensive impact on cytoskeletal integrity. Actin filaments are fundamental for maintaining cell–cell junctions, controlling cell polarity, and directing organelle movement [25, 26]. The significant decrease in β‐actin is particularly relevant given that elevated β‐actin levels correlate with increased invasiveness in various cancers [27]. Furthermore, the concurrent reduction in GAPDH expression, which facilitates actin polymerization and microtubule bundling [28]. This suggests a coordinated disruption of cytoskeletal support mechanisms.

Our immunofluorescence analyses demonstrated that actin and tubulin were distributed throughout both the cytoplasm and nucleus following MA1 treatment. Although this distribution deviates from their predominantly cytoplasmic localization, growing evidence indicates that cytoskeletal proteins can shuttle between the cytoplasm and nucleus in response to cellular stress, drug exposure, or oncogenic processes. Recent studies have further highlighted functional roles for nuclear actin [29, 30, 31] and tubulin [32, 33, 34], including their involvement in transcriptional regulation and structural organization. Hence, the redistribution observed in MA1‐treated cells likely reflects adaptive cellular responses associated with cytoskeletal reorganization, suggesting a broader impact of MA1 on intracellular structural dynamics. Nevertheless, the precise mechanisms underlying this relocalization remain unclear and require further investigation.

The observed reduction in both β‐catenin and c‐Myc in MA1‐treated cells suggests potential modulation of the Wnt/β‐catenin signaling axis. The c‐Myc proto‐oncogene is a well‐established downstream target of Wnt/β‐catenin signaling [35], and in complex with Max, regulates the transcription of genes involved in key cellular processes such as protein synthesis, metabolism, ribosome biogenesis, and cell proliferation [36, 37]. Therefore, the decrease in c‐Myc expression may contribute to the broader cellular effects associated with the MA1 treatment. However, as the activation status of the Wnt pathway was not directly assessed in this study, these findings should be interpreted as indicative of an association between MA1 treatment and downregulation of Wnt/β‐catenin signaling components, rather than evidence of causal inhibition or pathway dependency. Further investigation is required to determine whether modulation of Wnt/β‐catenin signaling plays a direct or indirect role in the observed molecular responses.

Our analysis of EMT markers revealed significant downregulation of vimentin and N‐cadherin following MA1 treatment. Vimentin reduction prevents tumor cell migration [38], while decreased N‐cadherin suggests potential reversal of the invasive phenotype [39]. The concurrent decline in β‐catenin and FAT4 expression further supports disruption of EMT pathways, with FAT4 functioning as a tumor suppressor [40]. These findings indicate that MA1 may effectively counter the metastatic potential of OC.

Contrary to expectations, MA1‐induced cytotoxicity involves minimal contribution from the intrinsic apoptotic pathway, as evidenced by reduced BAD and BAX expression. Our immunofluorescence analyses further revealed staining patterns indicating both cytoplasmic and nuclear localization of BAX. Although such distributions may initially appear atypical, accumulating evidence suggests that proteins such as Bax can undergo dynamic relocalization under specific conditions, including cellular stress, drug treatment, and oncogenic transformation. Notably, BAX has been reported to translocate to the nucleus in response to apoptotic or stress‐related stimuli [41, 42]. The diffuse nuclear staining observed following MA1 treatment may therefore reflect treatment‐induced cellular stress and cytoskeletal remodeling, rather than a static subcellular localization. Overall, these patterns are indicative of altered cellular dynamics; however, further studies are required to elucidate the regulatory mechanisms underlying these changes.

The significant decreases in FADD, TRADD, and RIP1 levels, coupled with Bcl2 inhibition, indicated involvement of the extrinsic apoptosis pathway [43, 44]. The detection of cleaved caspase‐3, which has emerging roles in non‐apoptotic processes such as cytoskeleton remodeling [45], suggests MA1 triggers alternative pathways beyond traditional apoptosis. Our investigation revealed evidence for multiple cell death mechanisms operating simultaneously.

Autophagy is initiated by the formation of autophagic vesicles that mature into autophagosomes through the coordinated action of two key molecular complexes. The first complex includes PI3K, p150, and Beclin‐1, while the second comprises ATG5, ATG12, and LC3B‐I, the latter undergoing lipidation to form LC3B‐II. This processed LC3B‐II associates with the autophagosomal membrane, promoting autophagosome maturation and completion of the autophagic process [46, 47]. In the present study, the expression of autophagy‐related markers (ATG5, Beclin‐1, and LC3BI) was evaluated in MA1‐treated A2780S and A2780CP cells using western blotting and immunofluorescence. Western blot analysis showed no significant change in ATG5 expression following MA1 treatment, whereas a modest increase in Beclin‐1 and a marked increase in LC3BII levels were observed. These findings suggest enhanced autophagosome formation and implicate autophagy in MA1‐induced cytotoxicity. However, given the dynamic and complex nature of autophagy, increased LC3BII level should be interpreted as indicative of altered autophagic activity rather than definitive evidence of complete pathway activation. Ultrastructural analysis further supported these observations by revealing the presence of autophagic vacuole.

Immunofluorescence analysis demonstrated that ATG5 localized to both the cytoplasm and nucleus. Although ATG5 is traditionally associated with the cytoplasmic autophagy machinery, accumulating evidence indicates that it can translocate to the nucleus under stress conditions and participate in non‐canonical functions [48, 49]. Notably, ATG5 expression displayed a decreasing trend in MA1‐treated cells. Previous studies have shown that autophagy can proceed in the absence of ATG5 or ATG7 under specific stress conditions, indicating the existence of alternative, ATG5/ATG7‐independent pathways [50].

Collectively, these findings suggest that MA1 modulates autophagy through multiple mechanisms, including LC3B‐associated autophagosome formation and differential regulation of ATG5. The observed downregulation and nuclear localization of ATG5 may indicate a shift toward noncanonical autophagy pathways. These effects are cell line–specific, as supported by prior literature [51], reflecting distinct autophagic responses in cisplatin‐sensitive and resistant cells. The differential responses observed between A2780S and A2780CP cells highlight the context‐dependent nature of autophagy, which may lead to either cell survival or cell death depending on the cellular background. These observations suggest that MA1 may differentially influence autophagy‐related pathways in sensitive and resistant cells. These findings describe treatment‐associated alterations and do not represent a comprehensive analysis of autophagic flux or pathway dynamics. However, future studies will be required to elucidate the functional role of autophagy and to determine whether these alterations contribute directly to the observed cytotoxic effects.

Additionally, increased caspase‐1 levels demonstrate pyroptosis initiation, a pro‐inflammatory form of cell death with important implications for cancer immunotherapy [52].

Based on our findings, MA1 induces cytoskeletal destabilization, activating multiple interconnected cell death pathways. The depletion of cytoskeletal proteins leads to DNA damage, as indicated by increased γ‐H2AX levels. While classical apoptotic markers were downregulated, the autophagy markers and the pyroptosis marker (caspase‐1) were upregulated, indicating a shift from apoptotic cell death toward autophagic and pyroptotic pathways. These results suggest that MA1 promotes non‐apoptotic, stress‐induced cytotoxicity through mechanisms involving cytoskeletal degradation, DNA damage, and inflammatory responses. However, these observations are correlative, and further studies are required to elucidate the precise mechanisms underlying these responses.

To further support these experimental observations, molecular docking analysis was conducted with selected cytoskeletal, metabolic, and cancer‐related signaling proteins. MA1 exhibited the highest predicted binding affinities for key regulators of cytoskeletal stability, including vimentin and α/β‐tubulins, as well as for metabolic and cell survival proteins such as GAPDH. These predicted interactions provide a plausible molecular explanation for the cytoskeletal disruption and cytotoxic effects observed following MA1 treatment.

The comprehensive disruption of cytoskeletal integrity by MA1, combined with its ability to target multiple oncogenic pathways and activate diverse cell death mechanisms, positions it as a promising therapeutic candidate. The simultaneous targeting of structural and signaling components may overcome resistance mechanisms commonly observed with single‐target therapies. Furthermore, the activation of pyroptosis could potentially enhance immune recognition of treated cancer cells, providing additional therapeutic benefits.

## Conclusion

Our study reveals that MA1‐induced cytoskeletal disruption triggers a complex cascade of cellular responses leading to cancer cell death through multiple mechanisms. The compound's ability to simultaneously target cytoskeletal integrity, reverse EMT, disrupt key oncogenic pathways, and activate diverse cell death programs highlights the therapeutic potential of cytoskeletal‐targeting strategies in OC treatment. These findings provide a strong foundation for the further development of MA1 as a novel anticancer agent.

## Conflict of interest

The authors declare no conflict of interest.

## Author contributions

NAH was involved in investigation, methodology, writing the first draft; IAB was involved in conceptualization, project administration, writing—reviewing/editing; SHM was involved in investigation, methodology; SR was involved in methodology; RV was involved in methodology; HAR was involved in resources, software; SAB was involved in data curation, formal analysis, resources; SD was involved in data curation, formal analysis, resources; SIH was involved in data curation, formal analysis; BKT was involved in formal analysis, resources, writing—reviewing/editing; MAA‐K was involved in methodology; YT was involved in conceptualization, data curation, project administration, supervision, writing—reviewing/editing.

## Supporting information


**Fig. S1.** Full‐length western blots for Fig. 1A. MW, Molecular Weights.
**Fig. S2.** Normalized protein band intensity for Fig. 1A using imagej. Data represents the mean ± standard deviation of three independent experiments. Statistical significance was determined using Student's *t*‐test. *P < 0.05, **P < 0.01, ***P < 0.001.
**Table S1.** Quantification of IF intensity for Figs 2A,B, 3A,B, 4A–D.
**Table S2.** Normalized IF intensity for Figs 2A,B, 3A,B, 4A–D.
**Fig. S3.** Full‐length western blots for Fig. 6C. MW, Molecular Weights.
**Fig. S4.** Normalized protein band intensity for Fig. 6C using imagej. Data represents the mean ± standard deviation of three independent experiments. Statistical significance was determined using Student's *t*‐test. *P < 0.05, **P < 0.01, ***P < 0.001.
**Fig. S5.** Full‐length western blots for Fig. 10C. MW, Molecular Weights.
**Fig. S6.** Normalized protein band intensity for Fig. 10C using imagej. Data represents the mean ± standard deviation of three independent experiments. Statistical significance was determined using Student's *t*‐test. *P < 0.05, ***P < 0.001.
**Fig. S7.** Full‐length western blots for Fig. 12A. MW, Molecular Weights.
**Fig. S8.** Normalized protein band intensity for Fig. 12A using imagej. Data represents the mean ± standard deviation of three independent experiments. Statistical significance was determined using Student's *t*‐test. **P < 0.01.
**Fig. S9.** Full‐length western blot for Fig. 12C. MW, Molecular Weights.
**Fig. S10.** Normalized protein band intensity for Fig. 12C using imagej. Data represents the mean ± standard deviation of three independent experiments. Statistical significance was determined using Student's *t*‐test. *P < 0.05, **P < 0.01.
**Table S3.** Quantification of IF image for Figs 7–10E,F,12B,D.

## Data Availability

The data that support the findings of this study are available in the Supporting Information of this article.
