# Supplementary material for: Malformin A1–mediated cytotoxicity in ovarian cancer cells occurs through pyroptosis and autophagy
Source: FEBS Open Bio. 2026 Jul 4:10.1002/2211-5463.70296. Online ahead of print. doi: 10.1002/2211-5463.70296 (PMC13398916; doi:10.1002/2211-5463.70296)
Supplement: Supplementary file 1 — Fig. S1. Full‐length western blots for Fig. 1A. MW, Molecular Weights. Fig. S2. Normalized protein band intensity for Fig. 1A using imagej. Data represents the mean ± standard deviation of three independent experiments. Statistical significance was determined using Student's t‐test. *P < 0.05, **P < 0.01, ***P < 0.001. Table S1. Quantification of IF intensity for Figs 2A,B, 3A,B, 4A–D. Table S2. Normalized IF intensity for Figs 2A,B, 3A,B, 4A–D. Fig. S3. Full‐length western blots for Fig. 6C. MW, Molecular Weights. Fig. S4. Normalized protein band intensity for Fig. 6C using imagej. Data represents the mean ± standard deviation of three independent experiments. Statistical significance was determined using Student's t‐test. *P < 0.05, **P < 0.01, ***P < 0.001. Fig. S5. Full‐length western blots for Fig. 10C. MW, Molecular Weights. Fig. S6. Normalized protein band intensity for Fig. 10C using imagej. Data represents the mean ± standard deviation of three independent experiments. Statistical significance was determined using Student's t‐test. *P < 0.05, ***P < 0.001. Fig. S7. Full‐length western blots for Fig. 12A. MW, Molecular Weights. Fig. S8. Normalized protein band intensity for Fig. 12A using imagej. Data represents the mean ± standard deviation of three independent experiments. Statistical significance was determined using Student's t‐test. **P < 0.01. Fig. S9. Full‐length western blot for Fig. 12C. MW, Molecular Weights. Fig. S10. Normalized protein band intensity for Fig. 12C using imagej. Data represents the mean ± standard deviation of three independent experiments. Statistical significance was determined using Student's t‐test. *P < 0.05, **P < 0.01. Table S3. Quantification of IF image for Figs 7–10E,F,12B,D. [file FEB4-9999-0-s001.docx]

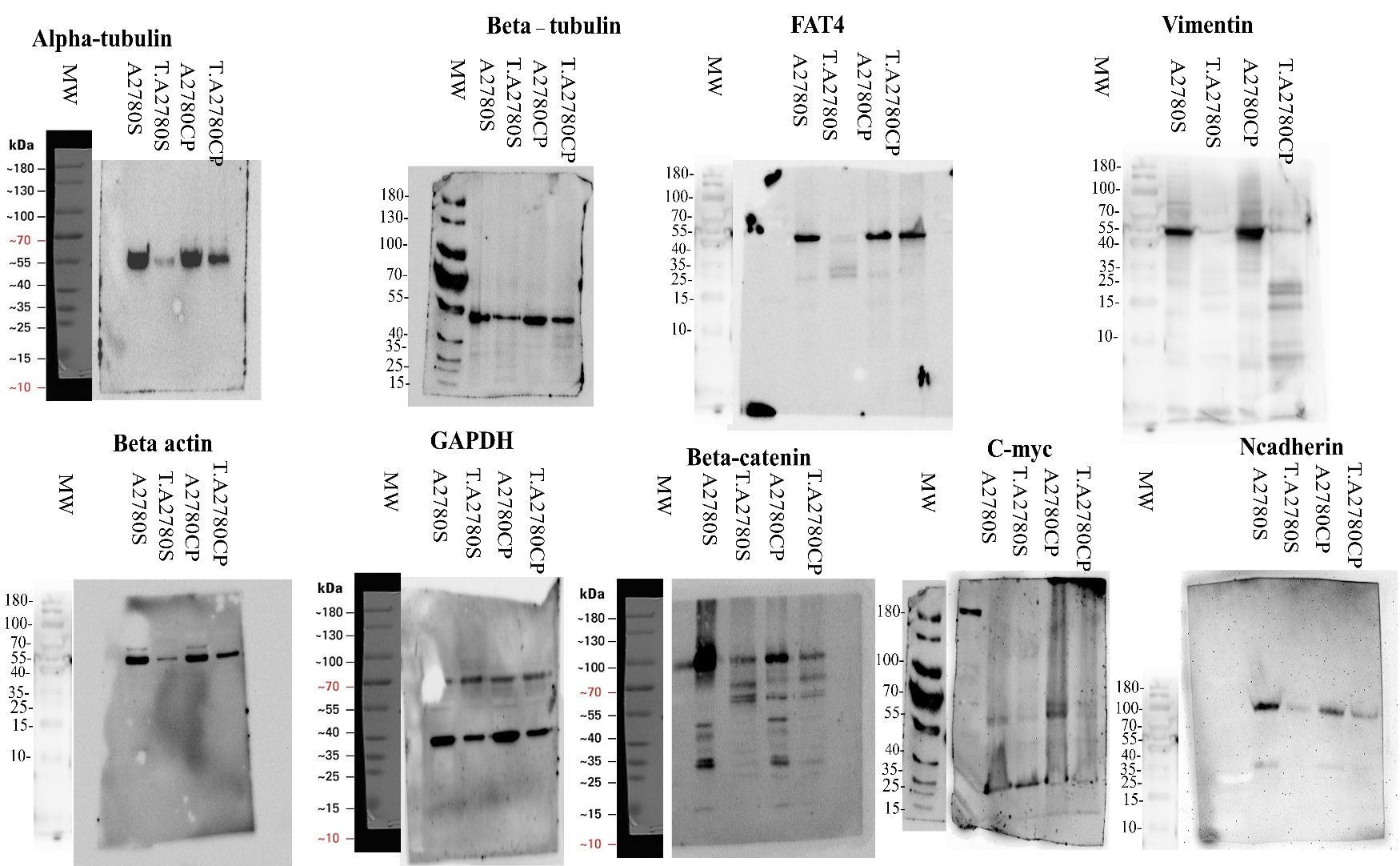


Figure S1: Full-length western blots for Fig.1a. MW: Molecular Weights.


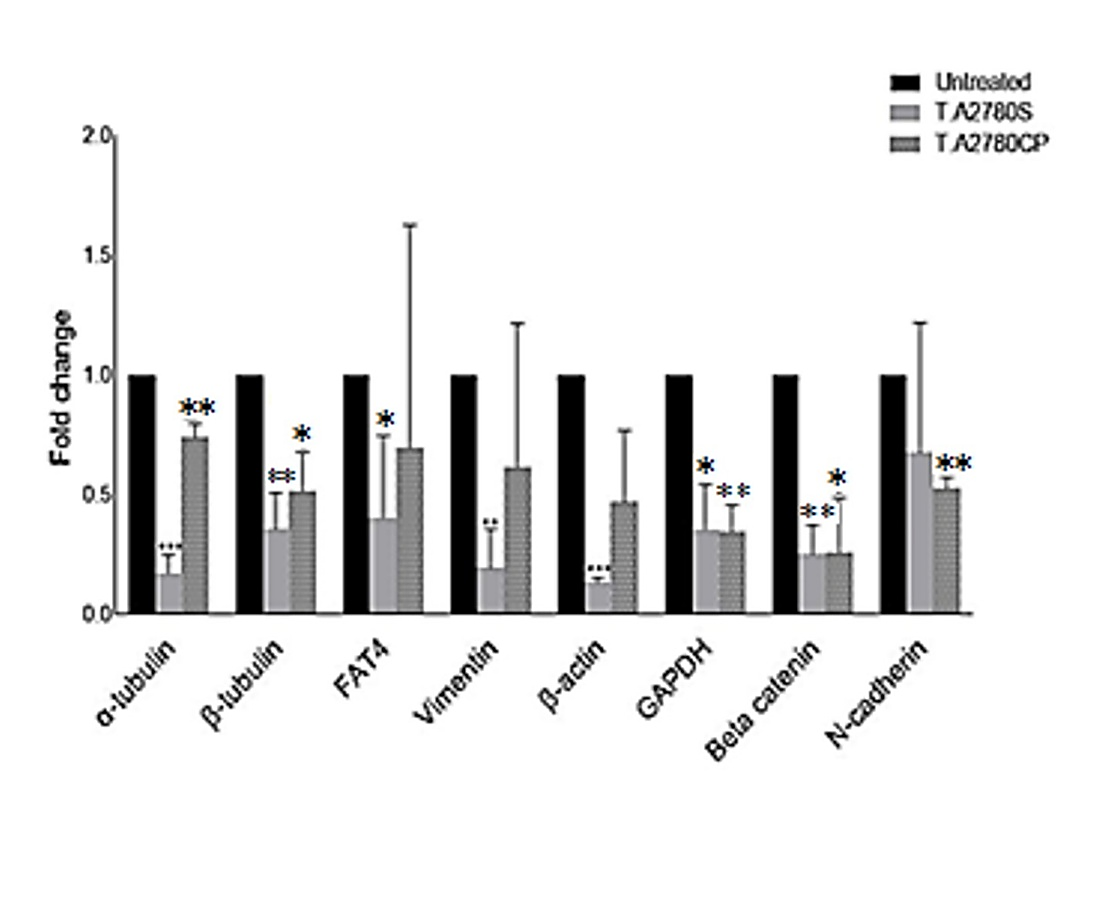


Figure S2: Normalized protein band intensity for Fig.1a using Image J. Data represents the mean ± standard deviation of three independent experiments. Statistical significance was determined using Student’s t-test. * P < 0.05, ** P < 0.01, *** P < 0.001.

Table S1: Quantification of IF intensity for Figures 2a, 2b, 3a, 3b, 4a, 4b, 4c, and 4d.

|  | **A2780S** | **T.A2780S** | **A2780CP** | **T.A2780CP** |
| --- | --- | --- | --- | --- |
| **Alpha-tubulin** | 11.741 | 2.31 | 4.733 | 1.476 |
| **Beta-tubulin** | 1.29 | .7 | 5 | .8 |
| **FAT4** | 3 | 1.631 | 9.430 | 9.380 |
| **Vimentin** | 2.8 | 2 | 6.2 | .837 |
| **Actin** | 14.7 | 2 | 1.909 | 1.4 |
| **GAPDH** | 5.2 | 1.9 | 1.634 | 1 |
| **Beta-catenin** | 2.1 | 1 | 2.6 | 1.4 |
| **N-cadherin** | 5.6 | 2.18 | 5.82 | 1.08 |

Table S2: Normalized IF intensity for Figures 2a, 2b, 3a, 3b, 4a, 4b, 4c, and 4d.

|  | **A2780S** | **T.A2780S** | **A2780CP** | **T.A2780CP** |
| --- | --- | --- | --- | --- |
| **Alpha-tubulin** | 1 | 0.196746 | 1 | 0.311853 |
| **Beta-tubulin** | 1 | 0.542636 | 1 | 0.16 |
| **FAT4** | 1 | 0.543667 | 1 | 0.994698 |
| **Vimentin** | 1 | 0.714286 | 1 | 0.135 |
| **Actin** | 1 | 0.136054 | 1 | 0.733368 |
| **GAPDH** | 1 | 0.365385 | 1 | 0.611995 |
| **Beta-catenin** | 1 | 0.47619 | 1 | 0.538462 |
| **N-cadherin** | 1 | 0.389286 | 1 | 0.186207 |


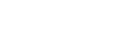


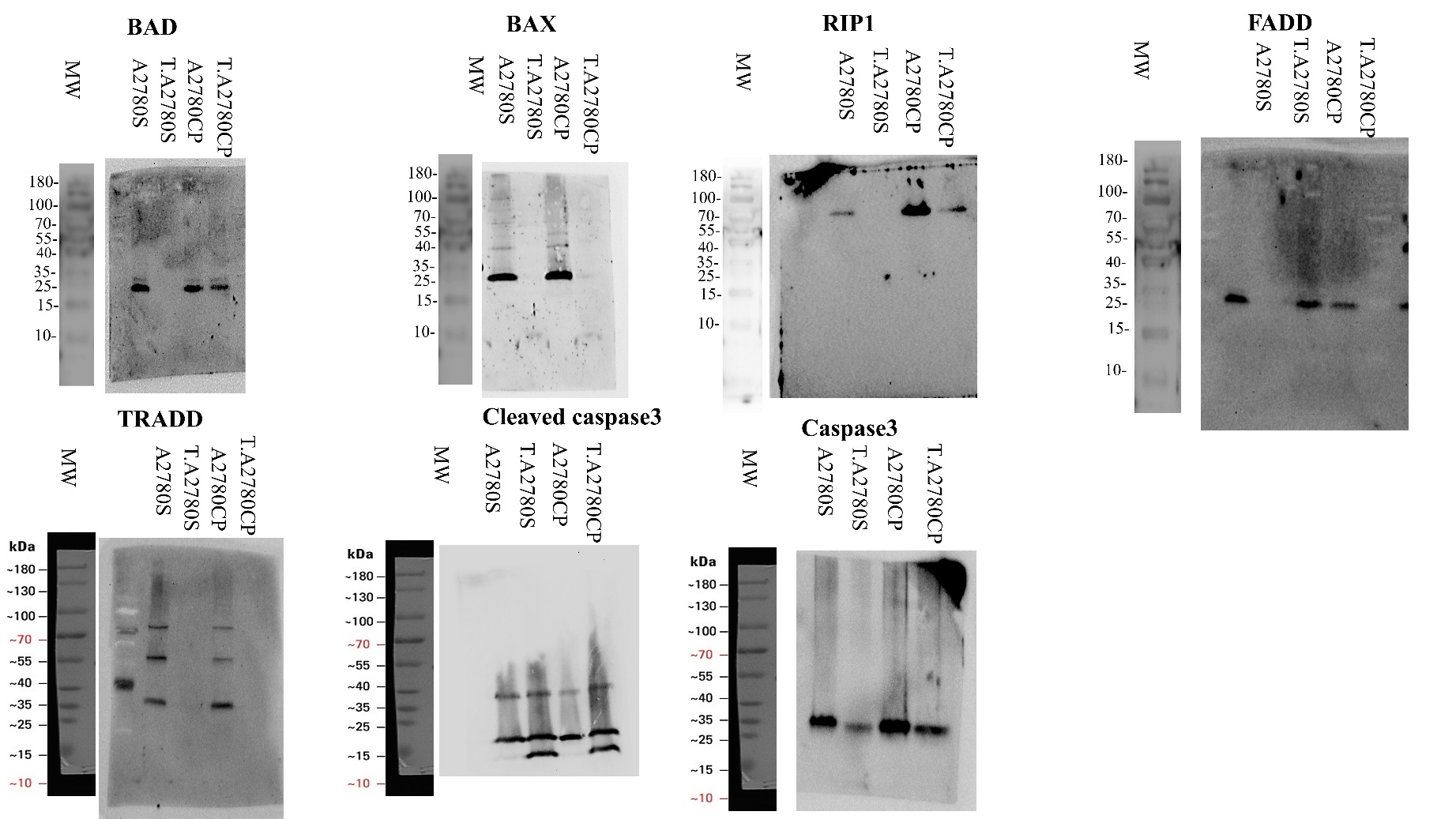


Figure S3: Full-length western blots for Fig.6c. MW: Molecular Weights.


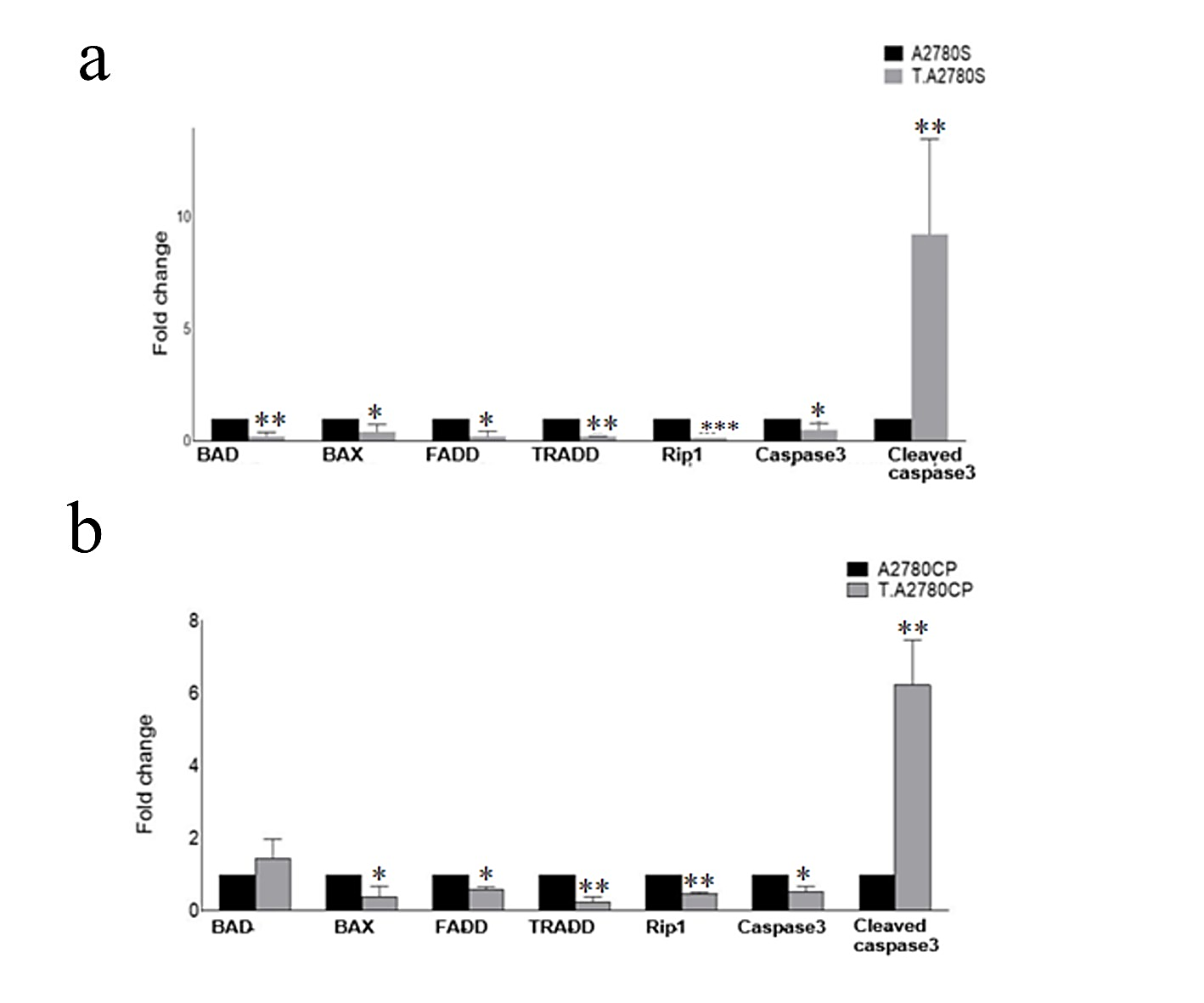


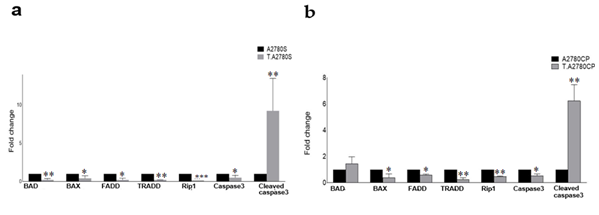


Figure S4: Normalized protein band intensity for Fig.6c using Image J. Data represents the mean ± standard deviation of three independent experiments. Statistical significance was determined using Student’s t-test. * P < 0.05, ** P < 0.01, *** P < 0.001.

**ATG5**

**Beclin1 LC3B**

T.A2780CP

A2780CP T.A2780S

A2780S

MW

T.A2780CP

A2780CP T.A2780S

A2780S

MW

T.A2780CP

A2780CP

T.A2780S

A2780S

MW


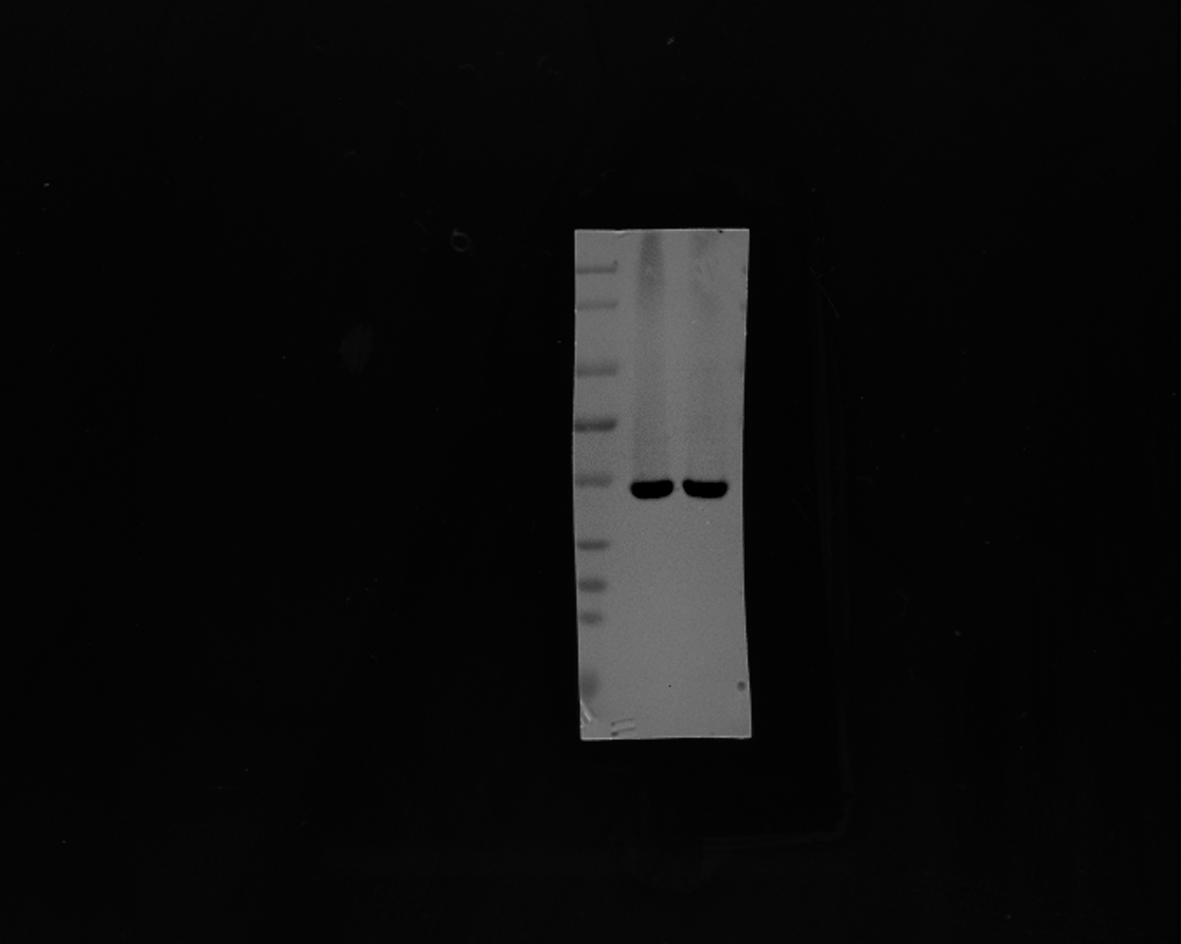


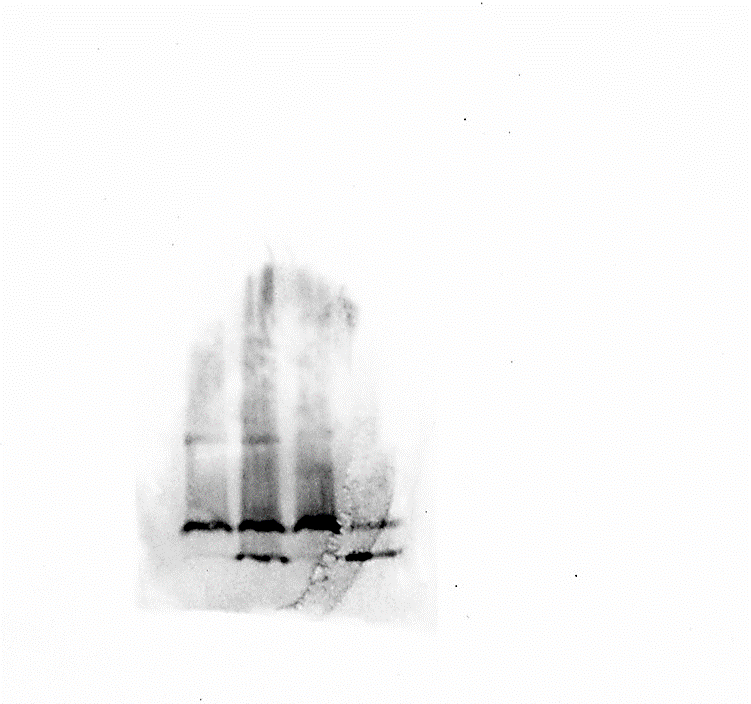

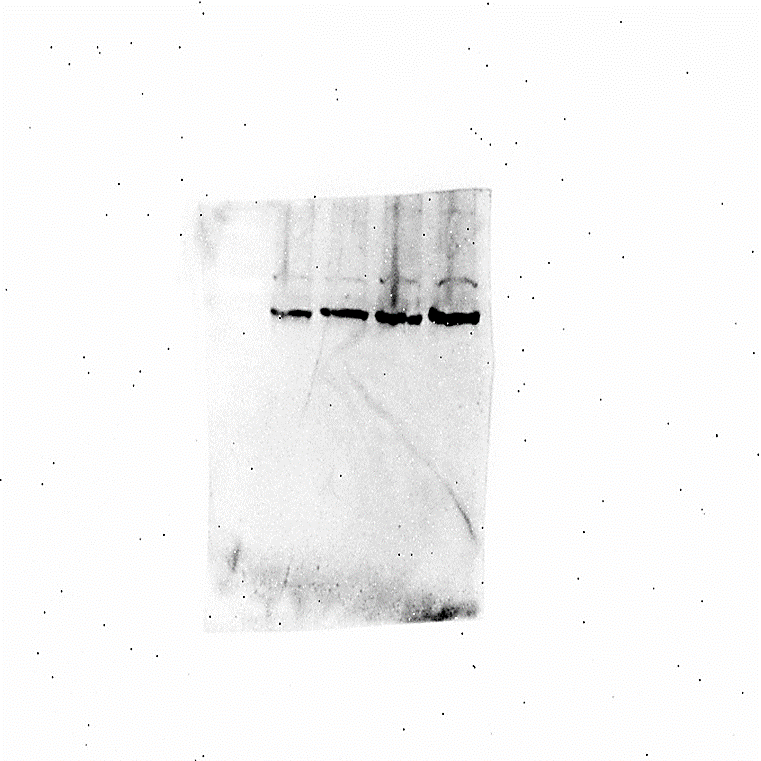

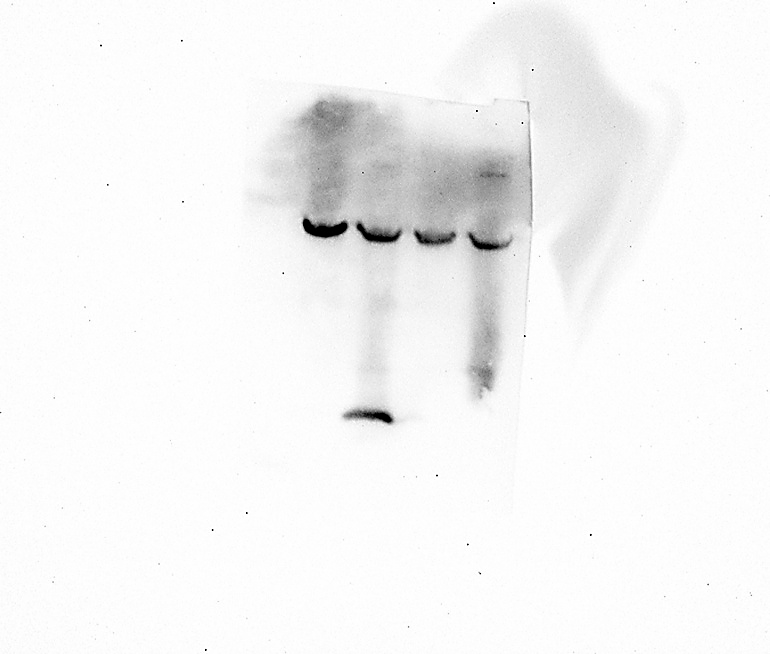


LC3B-I

LC3B-II

130-

100-

180-

70-

55-

40-

35-

25-

15-

10-

100-


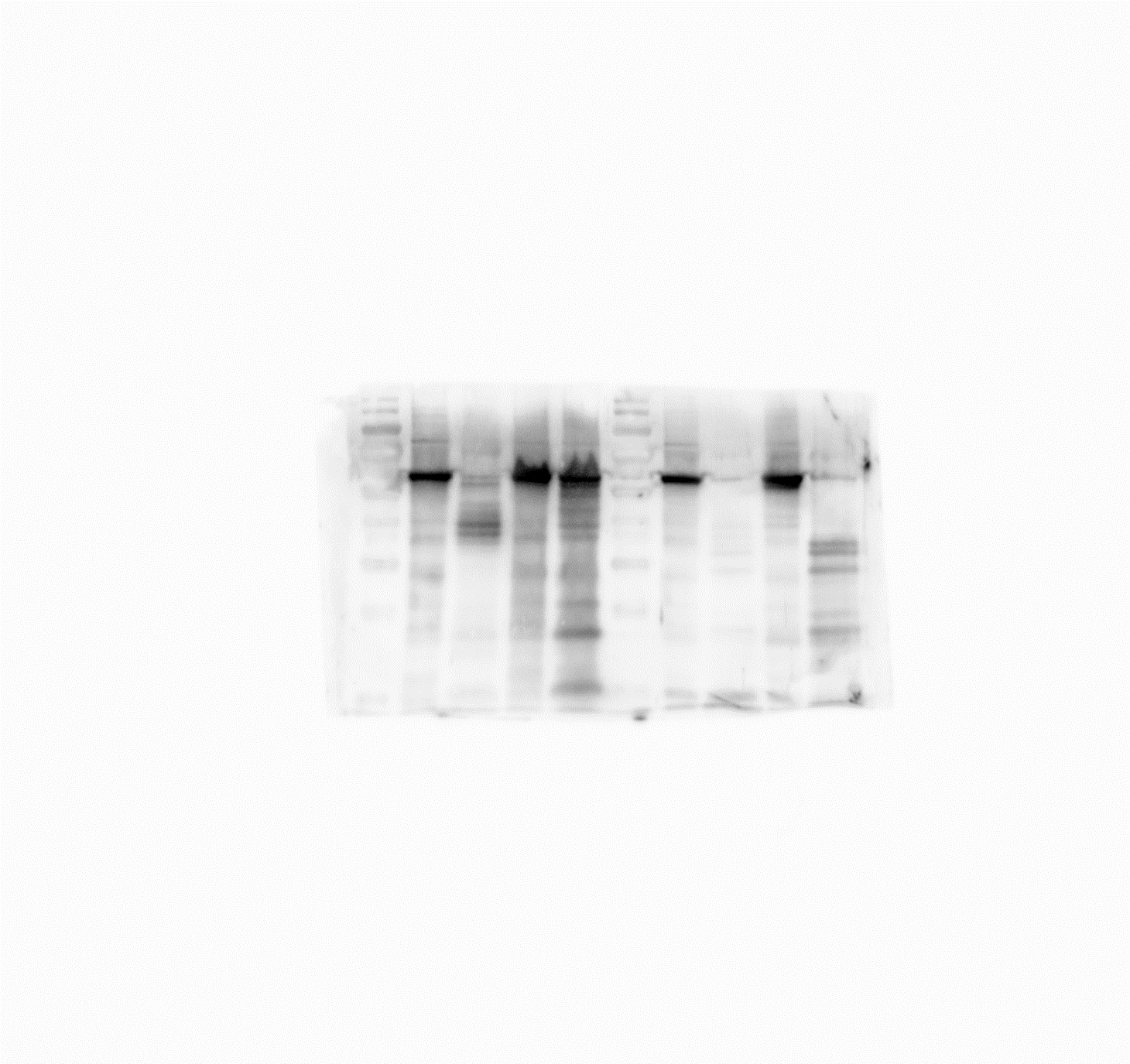


180-

70-

55-

40-

35-

25-

15-

10-

100-


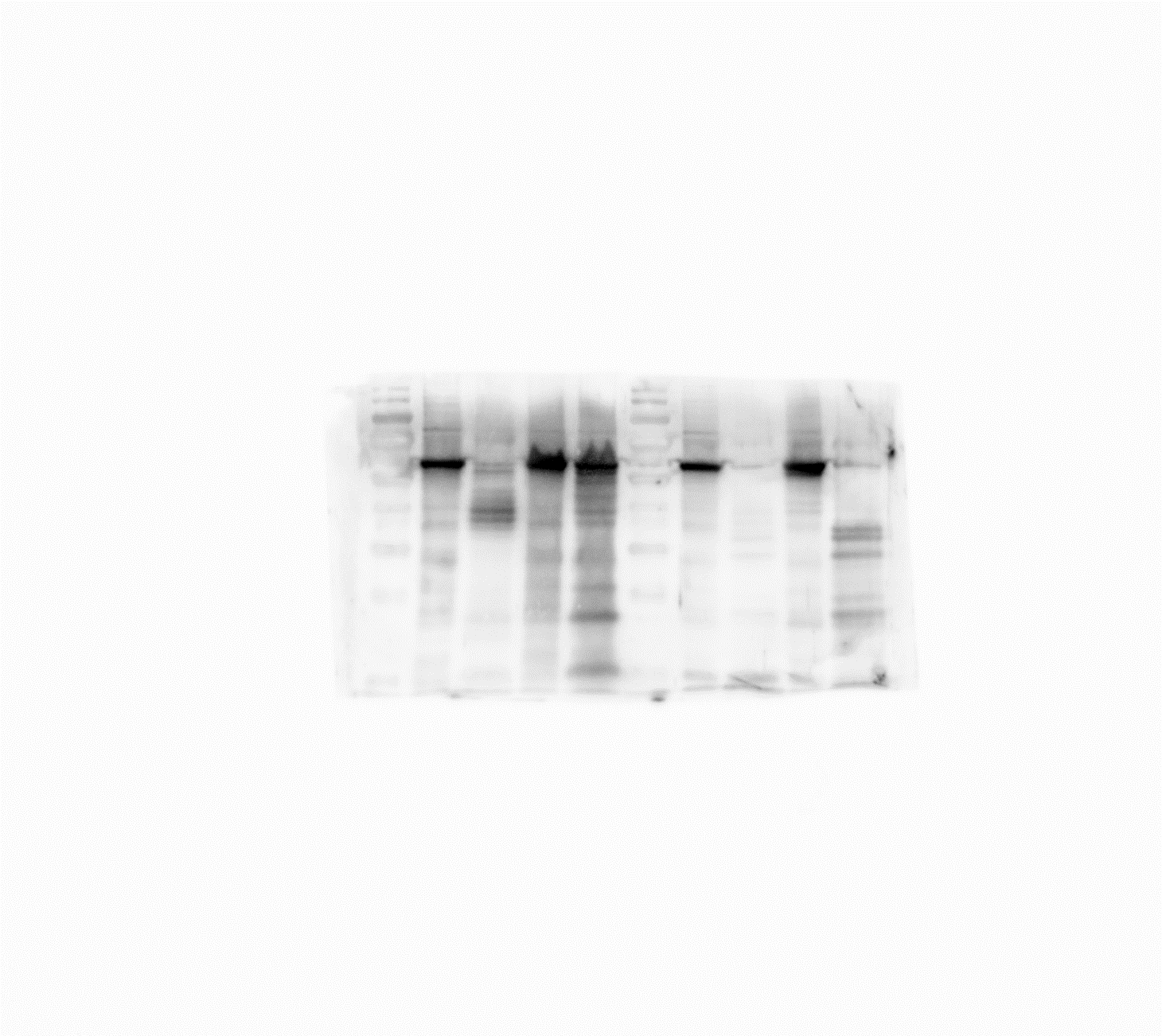


180-

70-

55-

40-

35-

25-

15-

10-

Figure S5: Full-length western blots for Fig.10c. MW: Molecular Weights.


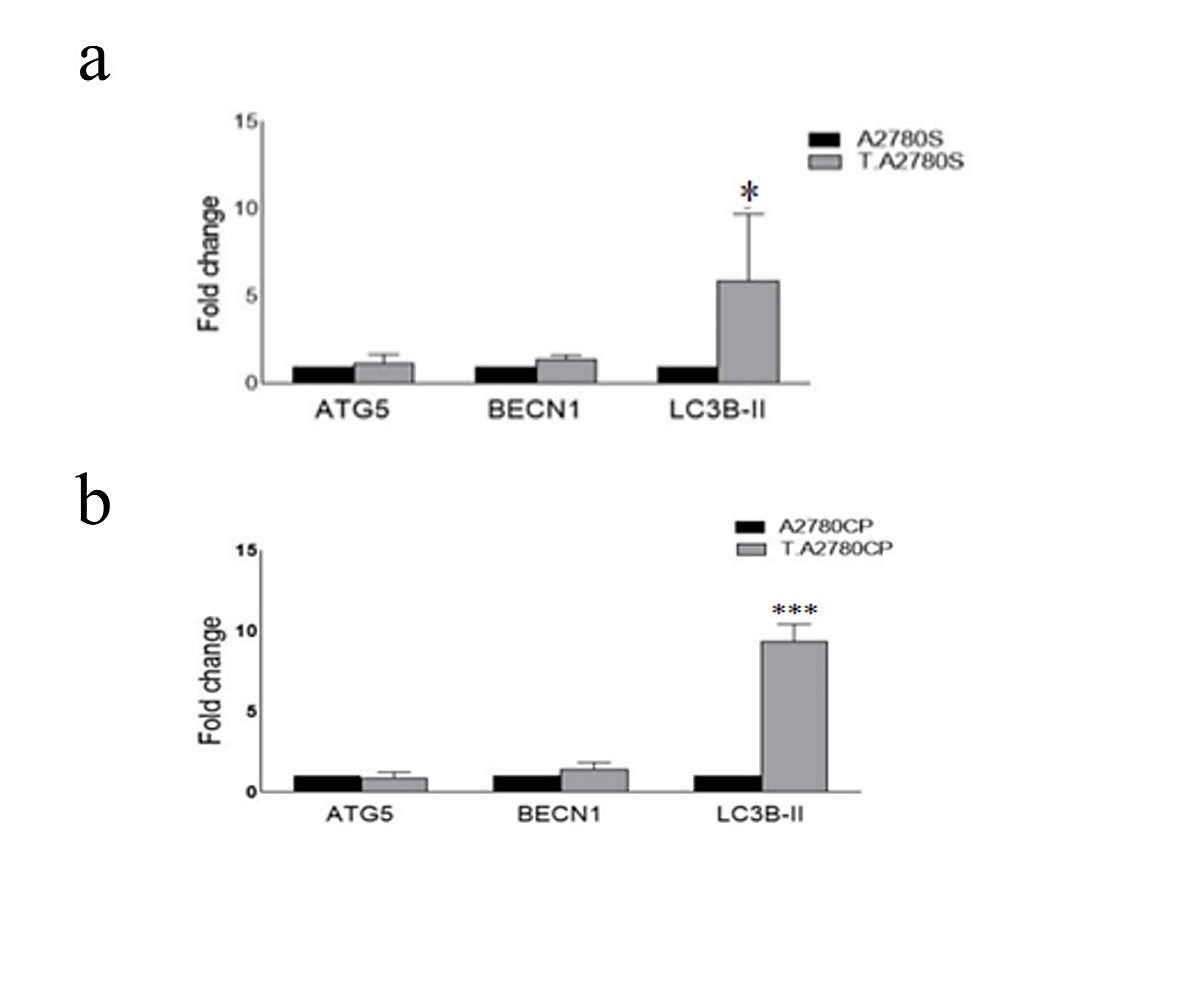


Figure S6: Normalized protein band intensity for Fig.10c using Image J. Data represents the mean ± standard deviation of three independent experiments. Statistical significance was determined using Student’s t-test. * P < 0.05, *** P < 0.001.

MW


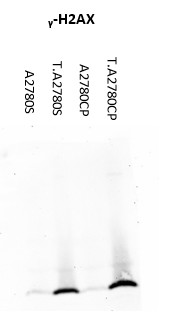


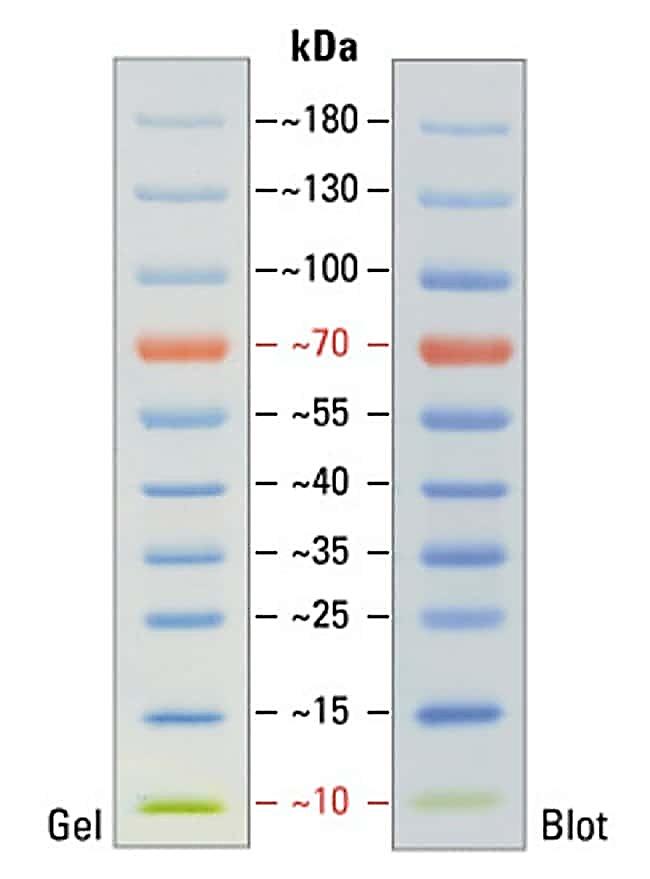

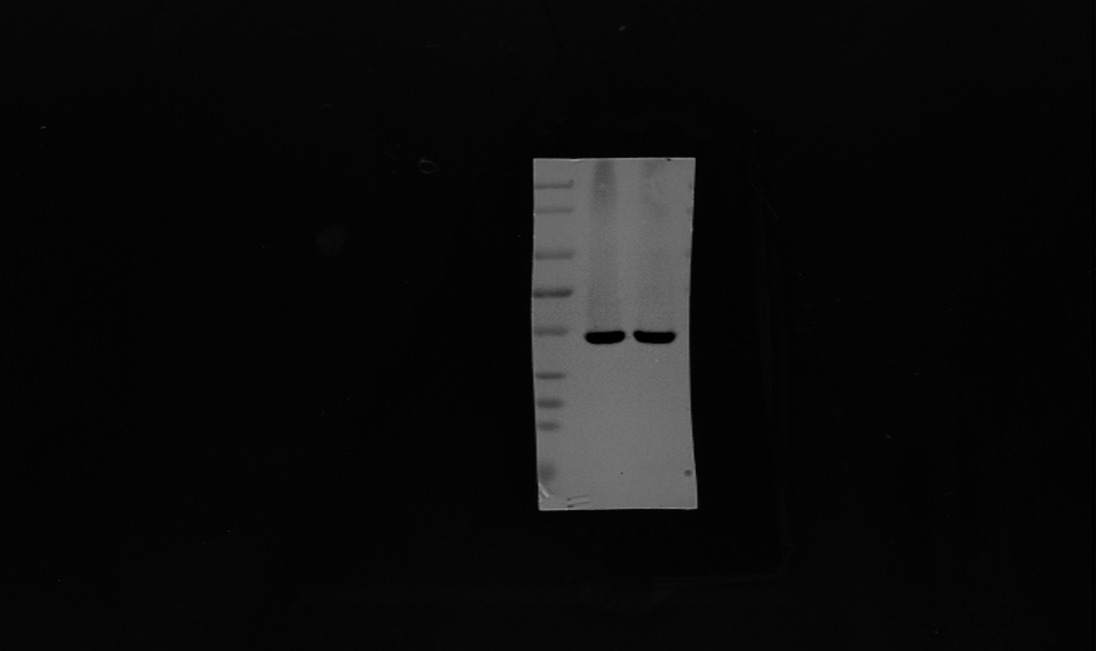

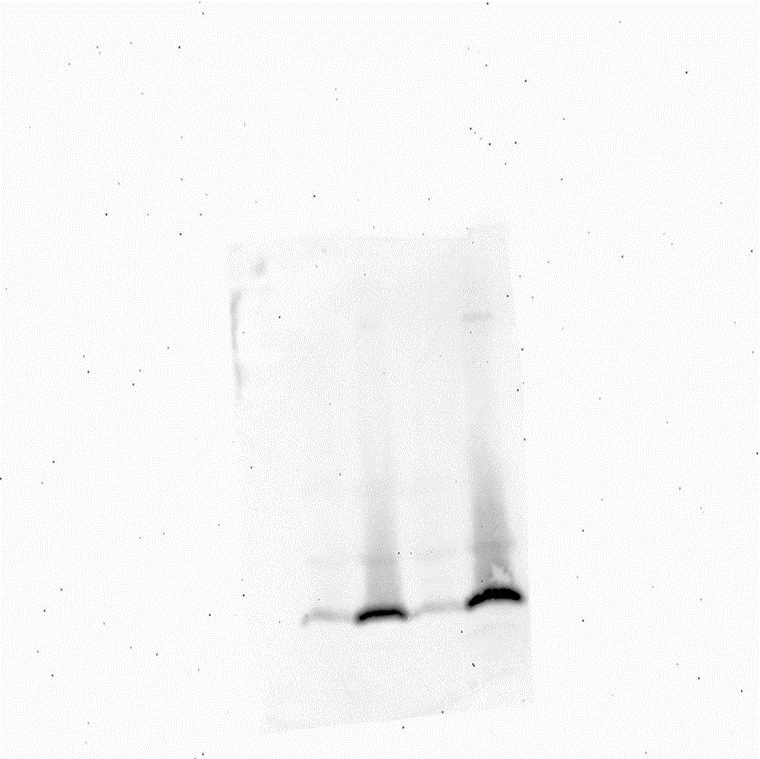


Figure S7: Full-length western blots for Fig.12a. MW: Molecular Weights.


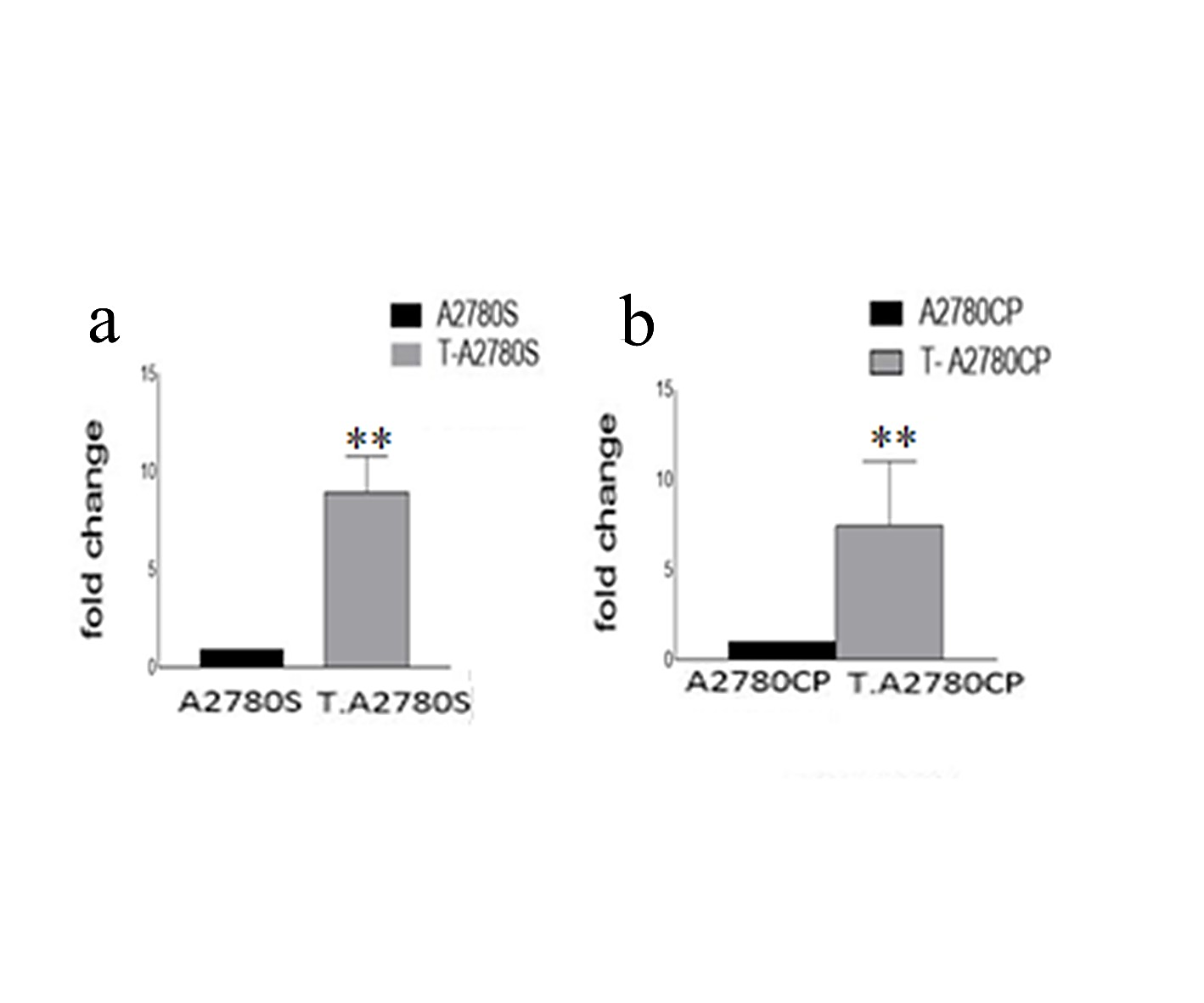


Figure S8: Normalized protein band intensity for Fig.12a using Image J. Data represents the mean ± standard deviation of three independent experiments. Statistical significance was determined using Student’s t-test. ** P < 0.01.


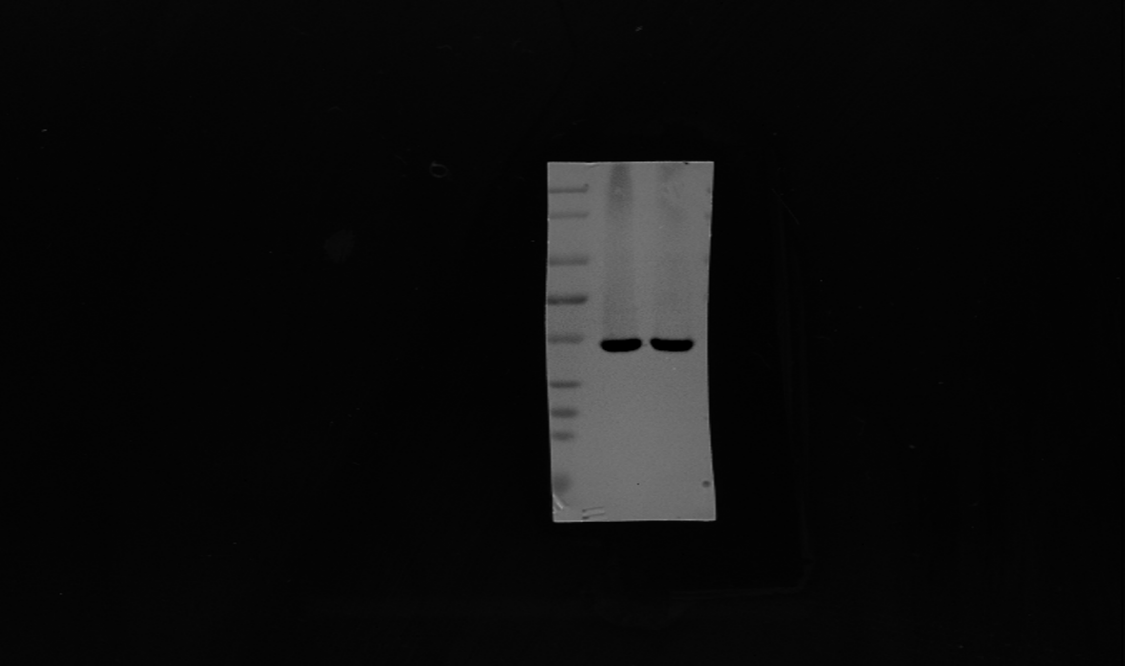

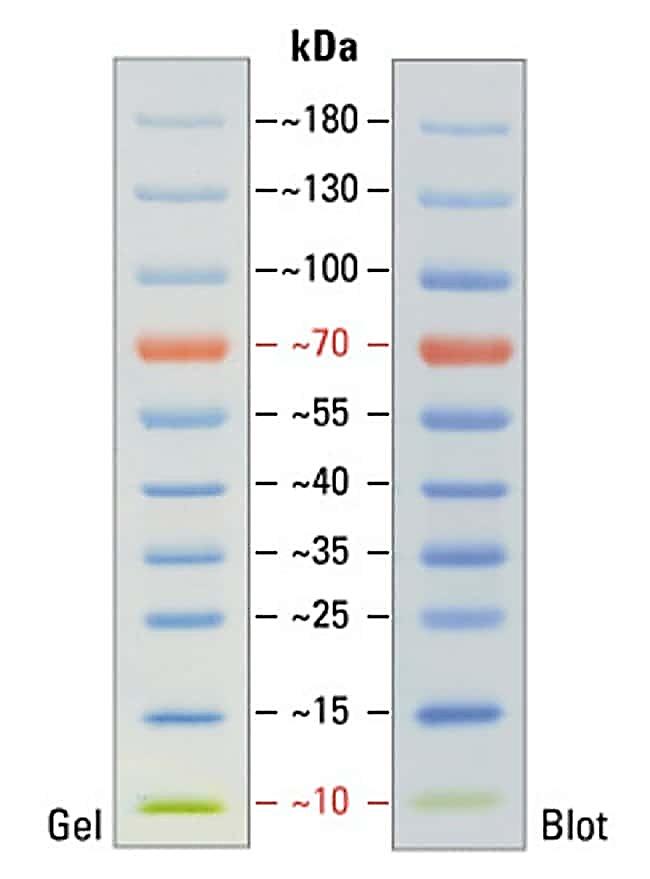


MW


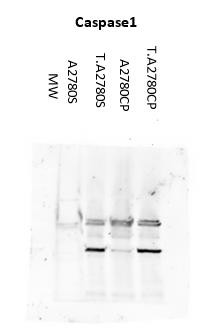


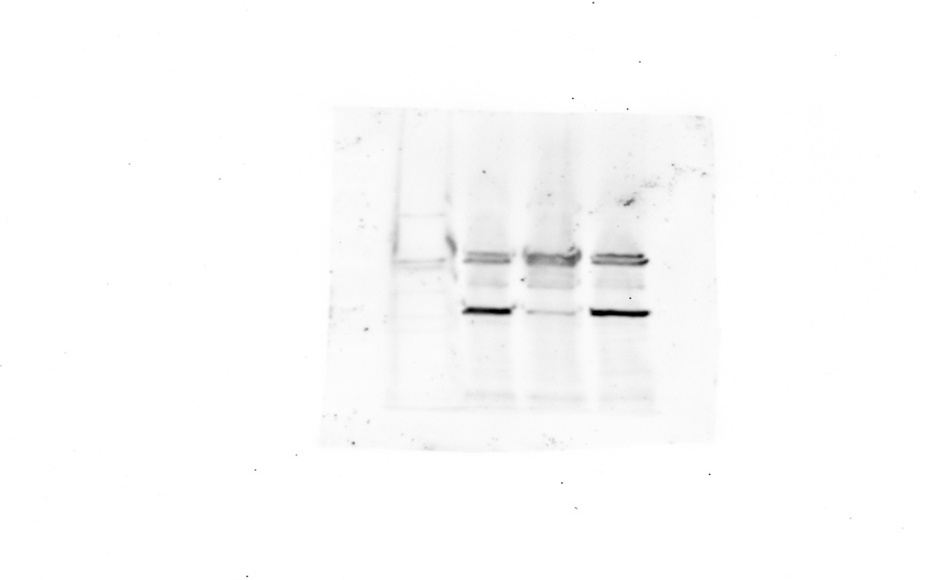


Figure S9: Full-length western blot for Fig.12c. MW: Molecular Weights.


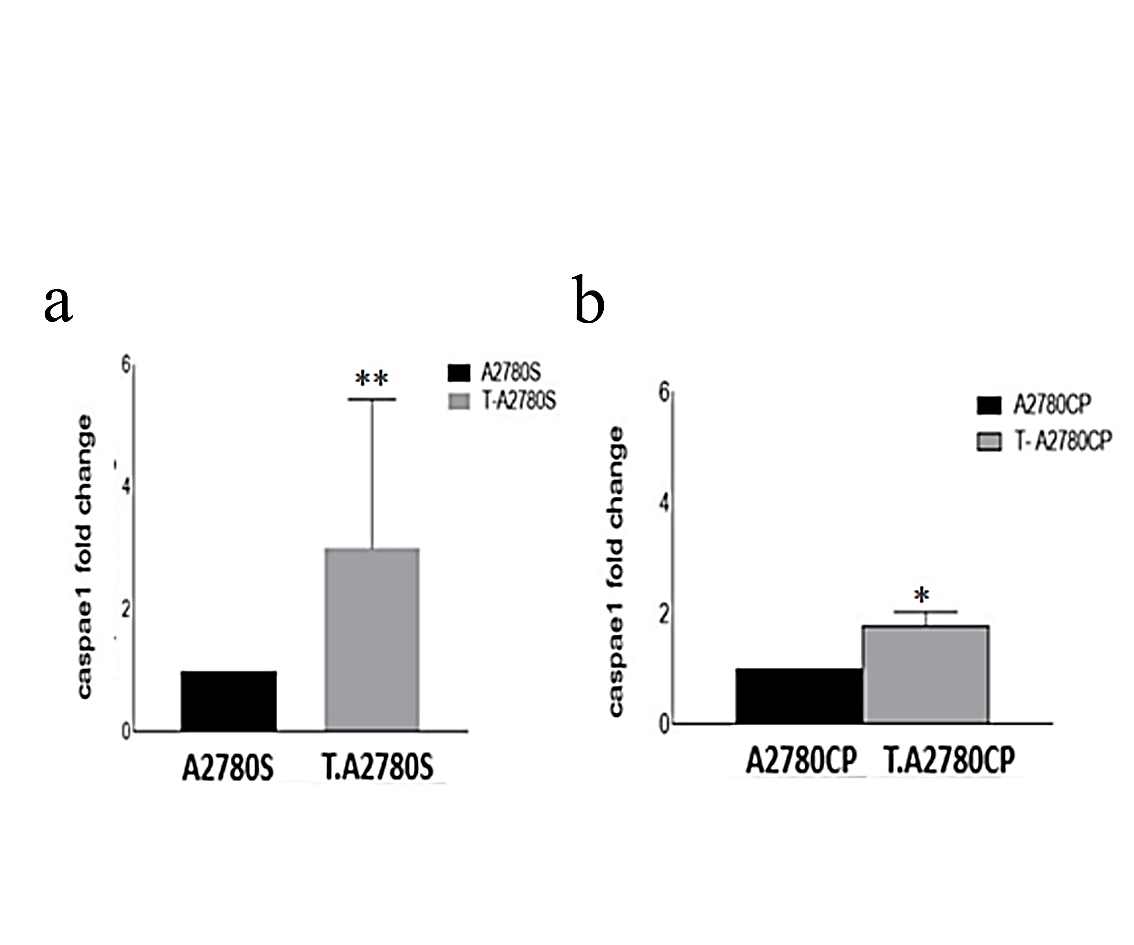

Figure S10: Normalized protein band intensity for Fig.12c using Image J. Data represents the mean ± standard deviation of three independent experiments. Statistical significance was determined using Student’s t-test. * P < 0.05, ** P < 0.01.

Table S3: Quantification of IF image for Fig.7, 8, 9, 10e, 10f, 12b, 12d.

|  | **A2780S** | **T.A2780S** | **A2780CP** | **T.A2780CP** |
| --- | --- | --- | --- | --- |
| **BAD** | 7.334 | 1.497 | 4.598 | 3.987 |
| **BAX** | 18.122 | 5.028 | 11.009 | 4.097 |
| **FADD** | 9.465 | 1.631 | 7.044 | 2.781 |
| **TRADD** | 11.315 | 2.143 | 7.735 | 3.269 |
| **RIP1** | 2.390 | 1.131 | 2.884 | 1.363 |
| **Caspase3** | 4.173 | .651 | 2.691 | 1.679 |
| **Cleaved**  **Caspase3** | .111 | 3.658 | 5.667 | 9.520 |
| **Atg5** | 13.402 | 11.933 | 7.888 | 5.730 |
| **Beclin1** | 8.755 | 7.743 | 3.384 | 4.318 |
| **Gamma-H2AX** | .077 | 4.465 | 1.082 | 6.530 |
| **Caspase1** | 2.567 | 8.967 | 4.394 | 7.168 |
